# Orthogonal dual-modification of proteins for the engineering of multivalent protein scaffolds

**DOI:** 10.3762/bjoc.11.88

**Published:** 2015-05-13

**Authors:** Michaela Mühlberg, Michael G Hoesl, Christian Kuehne, Jens Dernedde, Nediljko Budisa, Christian P R Hackenberger

**Affiliations:** 1 Forschungsinstitut für Molekulare Pharmakologie (FMP), Robert-Roessle-Str. 10, 13125 Berlin, Germany; 2 Freie Universität Berlin, Institut für Chemie und Biochemie, Takustr. 3, 14195 Berlin, Germany; 3 Technische Universität Berlin, AK Biokatalyse, Institut für Chemie, Müller-Breslau-Str. 10, 10623 Berlin, Germany; 4 Charité - Universitätsmedizin Berlin, Institut für Laboratoriumsmedizin, Klinische Chemie und Pathobiochemie, Augustenburger Platz 1, 13353 Berlin, Germany; 5 Humboldt Universität zu Berlin, Institut für Organische und Bioorganische Chemie, Institut für Chemie, Brook-Taylor-Str. 2, 12489 Berlin, Germany

**Keywords:** chemoselectivity, dual protein modification, lectin, multivalency

## Abstract

To add new tools to the repertoire of protein-based multivalent scaffold design, we have developed a novel dual-labeling strategy for proteins that combines residue-specific incorporation of unnatural amino acids with chemical oxidative aldehyde formation at the *N*-terminus of a protein. Our approach relies on the selective introduction of two different functional moieties in a protein by mutually orthogonal copper-catalyzed azide–alkyne cycloaddition (CuAAC) and oxime ligation. This method was applied to the conjugation of biotin and β-linked galactose residues to yield an enzymatically active thermophilic lipase, which revealed specific binding to *Erythrina cristagalli* lectin by SPR binding studies.

## Introduction

The chemical modification of proteins has been developed to a core discipline in chemical biology with diverse applications in all areas of the life sciences, including pharmacology, biophysics, biotechnology and cell biology [1–4]. In addition to the use of chemical labeling methods to study structure and function of proteins in vitro and in vivo, chemoselective conjugation techniques are also used to functionalize artificial protein scaffolds, such as viral capsids [5–7]. Such templates have self-assembled hierarchical structures that allow the generation of nanostructured scaffolds with precisely defined dimensions and configurations [7–12]. We have recently contributed to this field using globular proteins as multivalent scaffolds for the structurally-defined presentation of ligands. In a proof-of-principle study to engineer multivalent glycoprotein conjugates, we have used the incorporation of non-canonical amino acids (NCAA) [13] by supplementation based incorporation (SPI) [14–17] in auxotroph expression systems followed by the chemoselective Cu-catalyzed azide–alkyne cycloaddition (CuAAC) to attach carbohydrate ligands to the protein barstar [18].

In the current study, we aimed to extend this approach to the dual modification of proteins using a combination of two chemoselective, orthogonal conjugation reactions for the introduction of glycan ligands and biotin to a protein. Our main objective in this paper was the development of a robust synthetic methodology that allows the site-specific attachment of two distinct chemical modifications to a given protein, which can be used to target multivalent interactions. As a protein scaffold we selected the thermophilic lipase from *Thermoanaerobacter thermohydrosulfuricus* (TTL), since this protein is tolerant to high temperatures, a variety of solvents and other additives, and an enzymatic assay is available as a control for retained protein integrity and catalytic function [19].

Dual labeling techniques in protein synthesis are dependent on the availability of unnatural protein expression methods to install orthogonal chemical handles for subsequent biorthogonal modification reactions [20–21]. For instance, the groups of Chin, Liu and Lemke introduced two mutually compatible chemical handles by combining nonsense and/or quadruplet codon suppressions [22–25]. Although recombinant expression strains have been engineered to improve incorporation efficiency [26–28], double labeling approaches by nonsense or quadruplet codon suppression are often coping with low protein yields. The main reasons for these low yields are the competition of NCAA incorporation with translational frame shifting or termination, and low catalytic efficiency of engineered aminoacyl-tRNA synthetases [29].

Certainly, the most straightforward approach to achieve the dual modification of proteins is to combine unnatural protein expression with the site-directed modification of canonical amino acids, particularly cysteine. For example, SPI was used to introduce a NCAA such as azidohomoalanine (Aha) in a methionine-(Met)-auxotroph in combination with the chemical modification of the natural amino acid cysteine [30–31]. These handles were, e.g., addressed by CuAAC and disulfide bond formation, respectively, to introduce two distinct modifications. In addition also amber suppression for the installation of a ketone-containing NCAA (Ac-Phe) was combined with Cys-labeling for a site-specific FRET-labeling of proteins [32]. Despite these advances, the chemical modification of cysteine has some drawbacks including the high tendency for disulfide bond formation or cross reaction with other cysteine residues, reaction reversibility, and occasionally side-reactions with basic side chains, e.g., lysines [33].

Specifically, in the current paper we use in the current paper the oxime ligation [34–35] as the second orthogonal conjugation reaction in addition to CuAAC for the attachment of functional moieties to Aha residues installed by auxotroph expression. In order to install a second unnatural functionality in the protein, in addition to SPI, we utilized the well-established oxidative aldehyde formation at the *N*-terminus with NaIO_4_ [36–41]. With this approach, we aimed to engineer an artificial lectin-binding protein via chemical installation of several galactose moieties by CuAAC [18]. The second functionalization site at the protein’s *N*-terminus was conjugated with biotin using oxime ligation, by which the protein scaffold was immobilized on a streptavidin gold chip to monitor carbohydrate–protein binding studies by surface plasmon resonance (SPR). This immobilization strategy allowed easy handling and reproducible orientation, which are notable improvements over the alternative active ester immobilization. Although not directly demonstrated in the current paper, our approach required considerably lower amounts of the inhibiting glycoconjugate in comparison to the reverse approach, which involves immobilization of lectin and titration of the binder.

## Results and Discussion

### Protein design

Aha labelled TTL variants were always expressed with the SPI approach. Aha is a Met analogue and incorporation leads to full substitution of all Met residues in TTL by Aha residues. Six of the ten Met positions are solvent accessible (M1,M20, M21, M145, M150, M161) [42]. These positions are well distributed over the protein surface. In addition to the reasons stated in the introduction, the Met surface distribution made TTL an attractive choice for this proof-of-principle study to generate a double-functionalized protein scaffold for multivalent binding studies.

**Scheme 1:**
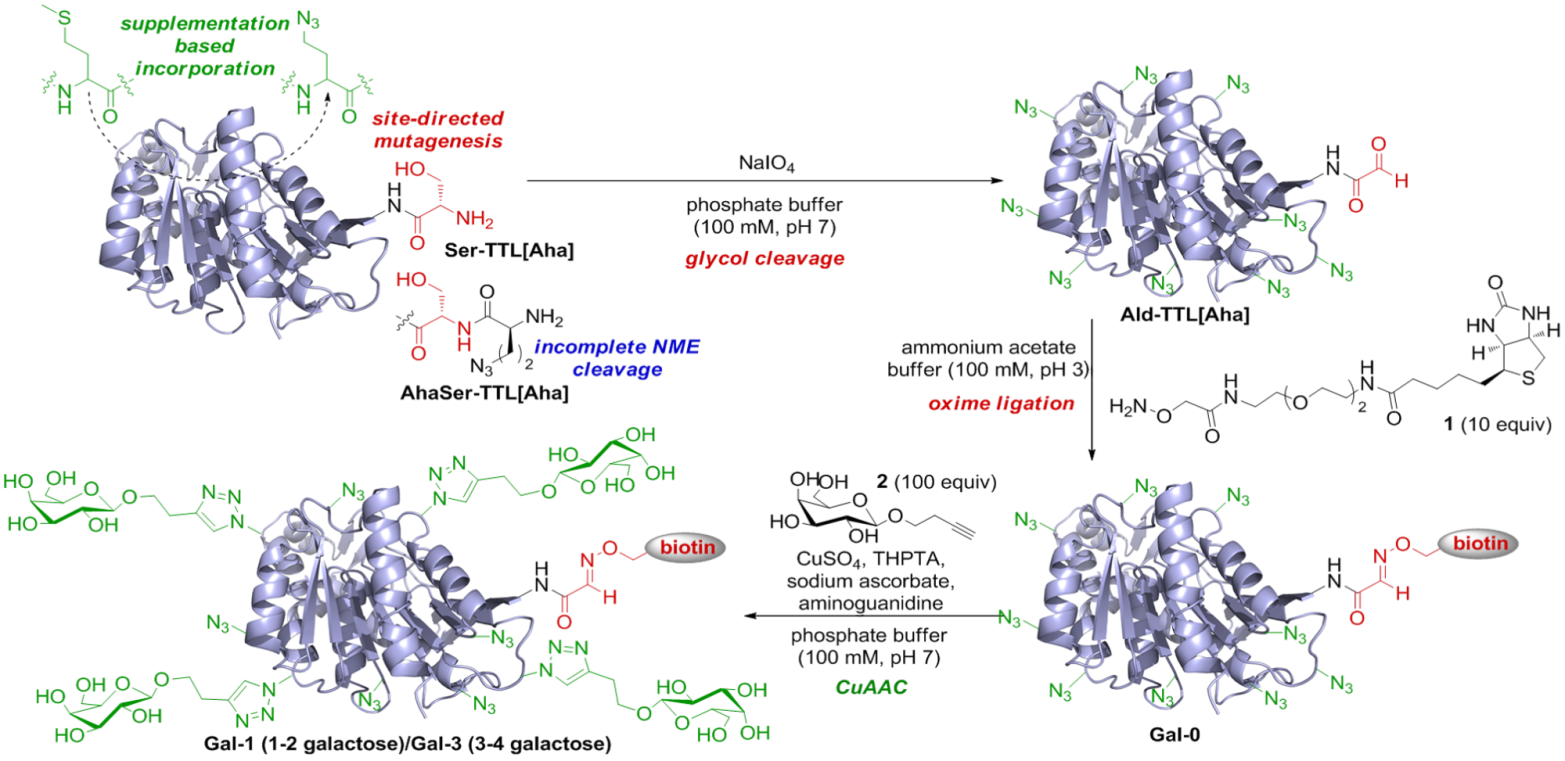
Protein design and dual-functionalization of TTL: periodate cleavage, oxime ligation and CuAAC.

In the beginning of our studies, we expressed TTL recombinantly with an *N*-terminal His-tag and tobacco etch virus protease (TEV) cleavage site, leaving an *N*-terminal Ser after the cleavage. However, we were unable to cleave the tag. This is probably due to structural constraints at the TTL’s N-terminus leaving the TEV protease recognition site inaccessible for the protease (for more information on protein design see Supporting Information File 1). Therefore, the construct was altered to contain an unmodified *N*-terminus with Ser at position 2. The *N*-terminal Met is cleaved when followed by small amino acids like glycine, alanine or serine in the native process of *N*-terminal methionine excision (NME) [43]. This process exposes Ser2 at the *N*-terminus for subsequent *N*-terminal oxime ligation. It has to be noted that the incorporation of Aha, as known [42,44], can hamper NME and therefore delivers in our case an approximate 1:1 mixture of TTL (estimated by MS, see Supporting Information File 1) with an *N*-terminal Ser (Ser-TTL[Aha]) and an *N*-terminal Aha (AhaSer-TTL[Aha]) together with nine additional Aha residues (Scheme 1). However, this *N*-terminal heterogeneity did not hamper our subsequent application, since only biotinylated protein could bind to the chip for SPR studies (see below).

### Dual-labeling of TTL

**Figure 1:**
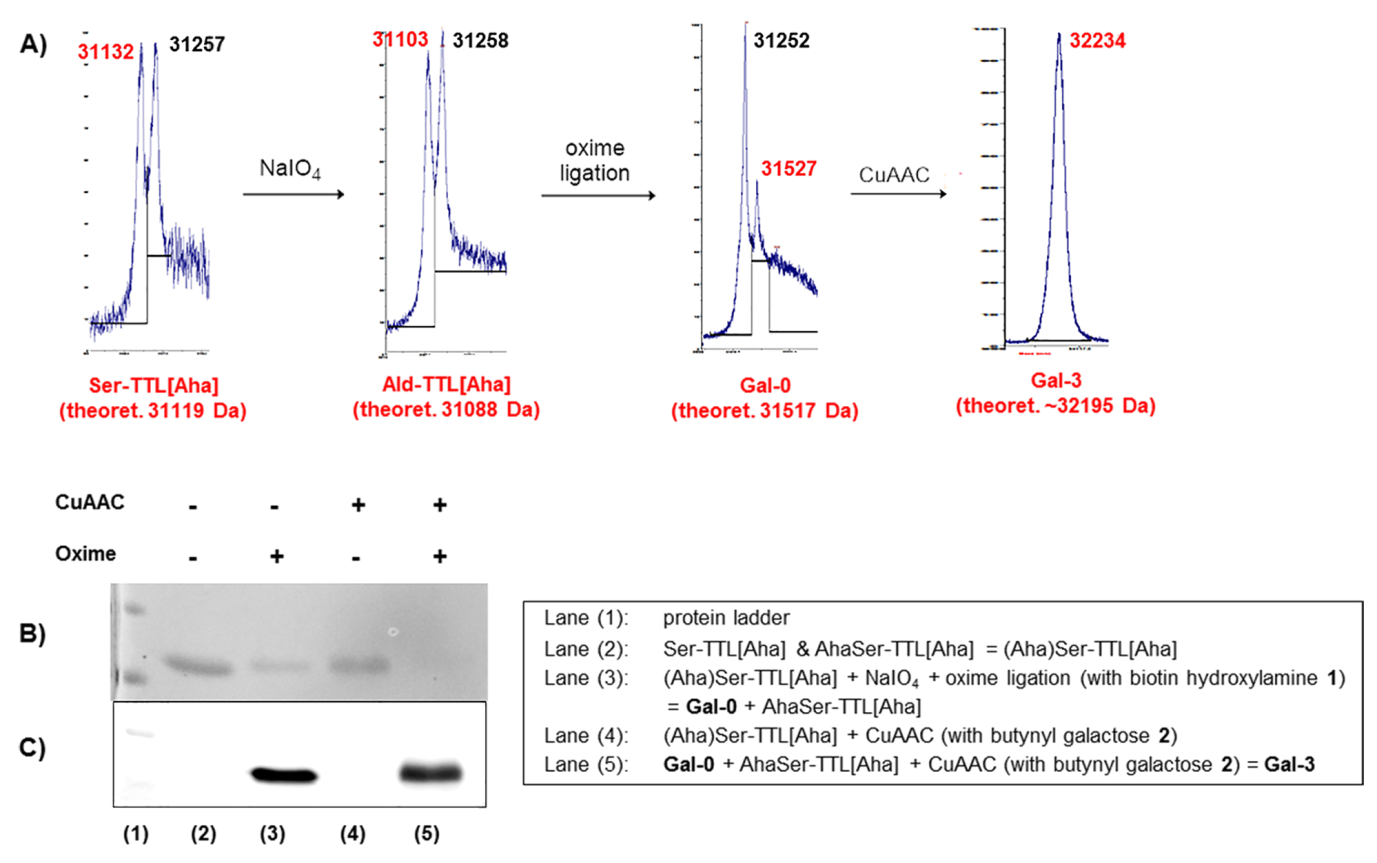
Dual-functionalization of TTL: A) MALDI–MS spectra (red: modified protein (as marked below); black: reference protein AhaSer-TTL[Aha]; *m*/*z* (calculated): [M+H]^+^ 31245 Da; for full spectra see Supporting Information File 1) B) SDS PAGE of TTL protein conjugates (Coomassie stain), C) Western Blot (streptavidin-peroxidase antibody).

Oxime ligation and CuAAC have been reported previously to be orthogonal to each other in DNA model systems and proteins obtained from amber and ochre suppression [24,45–46]. Since glycol cleavage is needed to generate the *N*-terminal aldehyde [39–40], we initialized our synthetic route with NaIO_4_ treatment since the galactose units installed by CuAAC would be efficient targets for a glycol cleavage, as shown previously [47]. Based on optimization experiments for the periodate treatment of *N*-terminal Ser peptides (data not shown), TTL was treated with sodium periodate in a phosphate buffer at pH 7 and 15 °C for 1 h and quenched with *N*-acetyl-Met to quantitatively form the aldehyde Ald-TTL[Aha] (Scheme 1, Figure 1A) [48]. For the oxime ligation with the synthesized biotin hydroxylamine derivative **1** (see Supporting Information File 1), several reaction conditions were screened to achieve full conversion based on MALDI–MS analysis for the Ald-TTL[Aha], in which the unreactive AhaSer-TTL[Aha] served as a reference point (Figure 1A), whereby it has to be noted that due to the limited resolution of the MALDI for proteins all detected mass values differ by a few Dalton from the theoretical masses, and the peak intensity for the functionalized biotinylated lipase (**Gal-0**) was usually lower in all MALDI spectra which was addressed to the lower detectability of **Gal-0** due to the attached biotin. Under rather mild reaction conditions at pH 7 with *p*-anisidine as a catalyst only 10% product was formed [49]. Lowering the pH and increasing the amount of hydroxylamine **1** promoted the desired Schiff’s base formation (see Supporting Information File 1) and full conversion to **Gal-0** could be achieved in an ammonium acetate buffer (100 mM, pH 3.0) with 20 equiv hydroxylamine **1**. The successful biotinylation could also be shown by SDS PAGE (sodium dodecyl sulfate polyacrylamide gel electrophoresis) and Western Blot analysis (see Figure 1B–C, lane 3).

To probe CuAAC, we first reacted the unmodified protein mixture (Aha)Ser-TTL[Aha] with the previously synthesized β-butynyl galactose **2** (Scheme 1). The conjugation reaction was performed in phosphate buffer (100 mM, 100 mM NaCl, pH 7) with varying amounts of CuSO_4_. Tris(3-hydroxypropyltriazolylmethyl)amine (THPTA), a good stabilizer for Cu(I) in solution [50], was applied in all coupling reactions and conversions were again checked by MALDI–MS, gel electrophoresis and Western Blot (Figure 1B,C, lane 4). As evidenced by MS-analysis, we could observe that both proteins Ser-TTL[Aha] (nine azides) and AhaSer-TTL[Aha] (ten azides) reacted with galactose alkyne **2** via CuAAC and different degrees of glycosylation could be achieved depending of the amount of Cu^2+^ applied in the reaction, though the maximum number of galactose units per protein that could be attached appeared to be five (data not shown). By applying a sequential oxime/CuAAC ligation protocol by applying CuAAC to the biotinylated protein **Gal-0**, we could show successful dual-functionalization of our protein. Again, depending on the Cu^2+^ concentration, different numbers of galactose units per protein could be achieved. Although a protein mixture of two proteins, bearing either nine or ten Aha residues which could potentially react with butynyl galactose **2**, the MALDI spectra of the final protein mixture after CuAAC showed surprisingly sharp peaks with a difference of only 1–2 galactose units, which might indicate that both proteins react to a similar degree with the alkynyl galactose **2** (see Supporting Information File 1). Lower concentrations of CuSO_4_ led to higher degrees of functionalization with 3–4 (10 mol %, **Gal-3**) galactose units, whereas higher amounts of Cu^2+^ led to lower degrees of functionalization with 1–2 clicked sugars (30 mol %, **Gal-1**). Further evidence for successful glycan attachment was provided by tryptic digest and MS/MS-analysis of **Gal-3**, which showed functionalization of two specific Aha residues (see Table S5 in Supporting Information File 1). It should be noted that higher concentrations of Cu^2+^ also led to precipitation and loss of protein material. Finally, the protein mixture was purified by centrifuge membrane filtration with a 100 mM phosphate buffer (100 mM NaCl, pH 7) to yield approximately 20–35% of the initial protein material (Aha)Ser-TTL[Aha] after dual-functionalization as judged by UV (see Supporting Information File 1).

### Stability and lectin binding studies

To ensure the stability of TTL throughout the dual-labeling process, we performed a lipase activity assay to demonstrate that the enzymatic activity could be retained. All protein samples thereby showed similar lipase activity, as determined by the colorimetric *p*-nitrophenol assay (see Supporting Information File 1).

**Figure 2:**
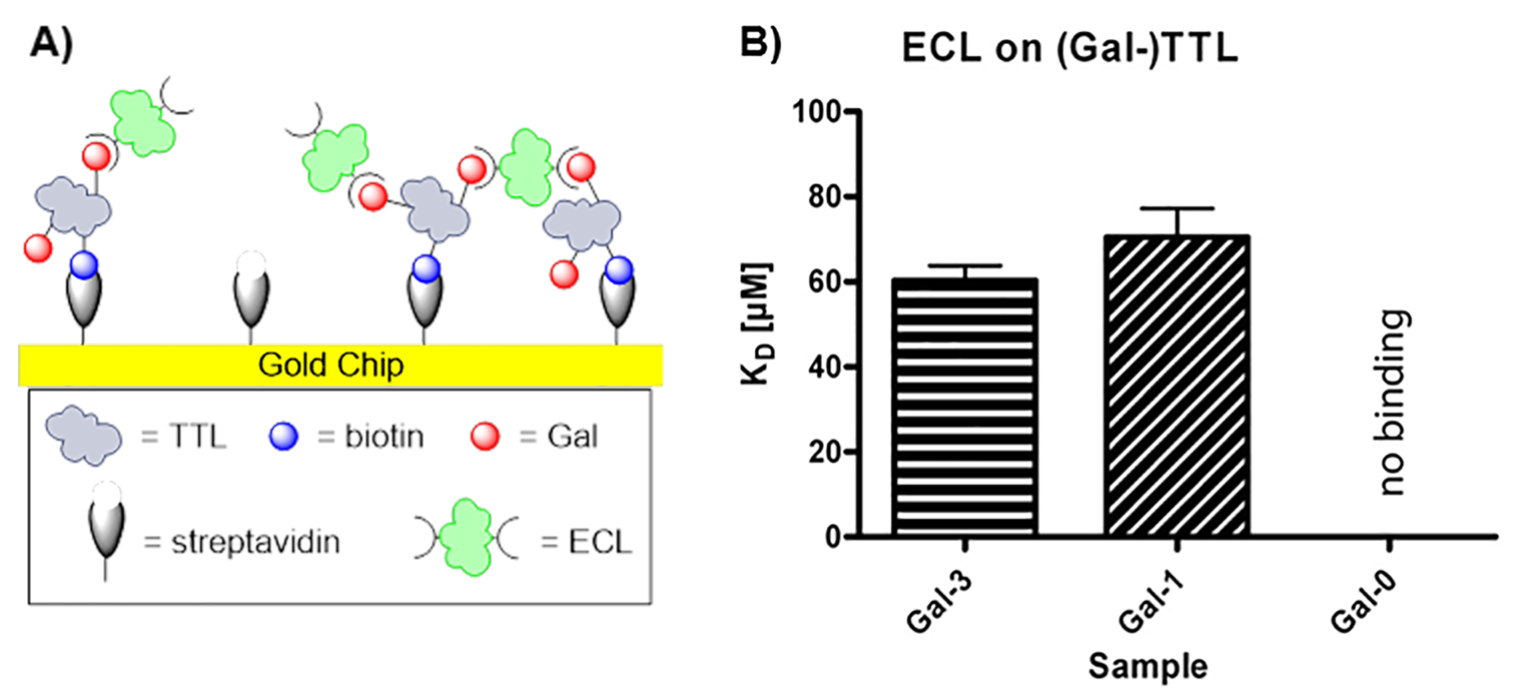
SPR measurements: A) set-up showing different binding events of the double-functionalized TTL to ECL; B) K_D_-data obtained for binding of **Gal-0**, **Gal-1** and **Gal-3**.

Finally, we also conducted surface plasmon resonance (SPR) studies to show the general applicability of our dual modified protein scaffold for measuring lectin binding constants (Figure 2 and Supporting Information File 1). We first probed the qualitative binding of *Erythrina cristagalli* lectin (ECL) to proteins **Gal-1** and **Gal-3** as well as **Gal-0** as a negative control. The three protein samples were each immobilized on a streptavidin-coated chip. Then, ECL was passed over the chip at different concentrations to determine the relative binding affinity for the immobilized glycosyl-TTL coated surface. At a concentration of 10 µM ECL, significant binding of both glycosylated protein samples towards the lectin were obtained (see Supporting Information File 1). The higher valent **Gal-3** revealed enhanced ECL binding, attributed to more frequent rebinding events. Also cross-binding of ECL to adjacent **Gal-3** proteins might occur due to the initial high immobilization level. In contrast, the non-glycosylated lipase exhibited no binding at all. To further characterize the binding efficiency, *K*_D_-values were determined by SPR measurements (for set-up see Supporting Information File 1). Again, for **Gal-0** no binding could be detected. Both glycosylated proteins, **Gal-1** and **Gal-3**, presented very similar and rather low *K*_D_-values (70 and 60 µM, respectively) with a slight tendency for stronger binding for the higher glycosylated protein **Gal-3** (see Supporting Information File 1). However, as the two Gal-binding sites of ECL are localized on opposite sides [51], our rather short butynyl linker might not be able to fully bend around the protein to achieve a multivalent effect [8,52], which might be the reason for the small difference between the two *K*_D_ values. In future experiments, different linker lengths should be probed to allow better binding of multiple carbohydrate units of one protein scaffold with multiple binding sites of one lectin molecule.

## Conclusion

In conclusion, we succeeded in the incorporation of two unnatural functional groups, namely azides and aldehydes, into a protein by combining a simple supplementation based incorporation and well-known oxidative periodate cleavage. To the best of our knowledge, this is the first successful combination of co-translational NCAA incorporation with post-translational periodate oxidation, which provides a novel tool to obtain a protein with two unnatural functional groups. For the functionalization of these unnatural moieties, we combined CuAAC with oxime ligation for the attachment of two different ligands, galactose and biotin, to the thermostable lipase TTL. The double functionalized TTL scaffold exhibited lectin binding properties while conserving its natural enzymatic activity, thereby demonstrating the principle applicability of this double protein functionalization strategy to the generation of new multivalent binding scaffolds.

Currently, we are further expanding our general dual-labeling strategy to other protein scaffolds as well as NCAAs to provide multiple distinct probes for the generation of individually designed protein binders. An important parameter in the future will be the combination with protein modelling as well as the implementation of different linker lengths between the protein and the binding units, to engineer precise protein models and study a variety of multivalent receptors.

## Experimental

**General protocol for glycol cleavage and oxime ligation on TTL.** A solution of the TTL (12 µM; 100 mM phosphate buffer, 100 mM NaCl, pH 7) was mixed with NaIO_4_ (3 equiv) and shaken for 1 h at 15 °C. *N*-Acetyl-Met (12 equiv) was added to the mixture and shaken for 1 h at 15 °C. The buffer was exchanged by centrifuge membrane filtration (14000 r/min). For the different buffers and catalysts see Table S1 (Supporting Information File 1). Biotin hydroxylamine **1** was added to the protein solution and the mixture was shaken overnight at 15 °C. For MALDI–MS analysis, the solutions were centrifuge-filtered (14000 r/min) and washed 4× with ammonium acetate solution (100 mM, pH 7) and 4× with ultrapure water. The proteins were analyzed by MALDI–MS measurements (Tables S3 and S4, Supporting Information File 1) and by SDS PAGE (Coomassie stain) and Western Blotting (streptavidin–peroxidase antibody, 1:1000) using a Mini-Protean Tetra cell system (BioRad) (see Figure 1).

For subsequent dual-functionalization, the samples were centrifuge-filtered with Dulbecco’s PBS buffer (100 mM, pH 7) after oxime ligation and directly applied in the CuAAC.

**General protocol for CuAAC on TTL.** A solution of the TTL (10 µM; 100 mM phosphate buffer, 100 mM NaCl, pH 7) was mixed with CuSO_4_ (1 M in 100 mM phosphate buffer, 100 mM NaCl, pH 7), sodium ascorbate (50 equiv to Cu^2+^) and 1-*O*-but-3-ynyl-α-galactopyranoside (**2**) (1100 equiv to protein), 80 µL THPTA (5 equiv to Cu^2+^), and aminoguanidine (8 mM) and shaken overnight at 15 °C. For the different CuSO_4_ concentrations see Table S2 (Supporting Information File 1). The solutions were centrifuge-filtered (14000 r/min) and washed 3× with buffer/EDTA-solution (100 mM phosphate buffer, 100 mM NaCl, 5 mM EDTA, pH 7) and 4× with ultrapure water. The proteins were analyzed by MALDI–MS measurements (Tables S3 and S4, Supporting Information File 1) and by SDS PAGE (Coomassie stain) and Western blotting (streptavidin–peroxidase antibody, 1:1000) using a Mini-Protean Tetra cell system (BioRad) (see Figure 1). Protein concentrations were checked by UV (λ = 280 nm).

**Lipase activity test** [53]. Lipase activity was determined by measuring the hydrolysis of *p*-nitrophenyl palmitate (*p*NPP; Sigma). Solution A (10 mM *p*-nitrophenyl palmitate in 10 mL ethanol) and solution B (100 mg gummi arabicum in 90 mL Tris-HCl buffer (50 mM, pH 8)) were mixed 1:9 and dispersed (ultraturrax, 3 min, 20000 min^−1^) to get solution C. For each measurement, 450 µL of solution C were mixed with 50 µL enzyme solution (0.13 nmol protein). The contribution of autohydrolysis was assessed by including a blank that contained the same volume of 50 mM Tris·HCl pH 8.0 instead of enzyme (background measurement). The samples were shaken at 50 °C for 1 h. Absorbance of released *p*-nitrophenol was measured at λ = 410 nm (Figure S10, Supporting Information File 1).

**Surface-plasmon-resonance (SPR).** SPR measurements were performed on a BiacoreX (GE Healthcare, Freiburg, Germany). Biotinylated TTL samples were coupled to streptavidin functionalized gold chips (SA-Chips, GE Healthcare, Freiburg, Germany). Before immobilization, the sensor chip was conditioned with three consecutive 1 min injections of 1 M NaCl and 50 mM NaOH.

For initial binding experiments, flow cell 2 (Fc2) of each chip was fully loaded (≈400 RU) with our protein. Flow cell 1 (Fc1) remained untreated and served as a reference. After immobilization, a sample volume of 100 µL of different concentrations of ECL solutions (1 or 10 µM) in HEPES buffered saline with calcium (HBS-Ca), 20 mM HEPES (4-(2-hydroxyethyl)-1-piperazineethanesulfonic acid), pH 7.4, 150 mM NaCl, 1 mM CaCl_2_ were injected over both lanes at a flow rate of 30 µL/min. The final binding signals were obtained by subtracting the resulting response units (RU) of the free reference lane from the data obtained for the sample lane (Fc2-Fc1, Figure S11, Supporting Information File 1). The association phase was followed by a 180 s dissociation phase. Washing and regenerating of both lanes was done by injecting 4 M MgCl_2_.

For *K*_D_ determination, chips were loaded to one third with the respective TTL and 50 µL ECL were injected in each run with a “wash after injection” step of 180 s for the dissociation phase, recording the response difference between ligand flow cell and reference flow cell. Washing and regeneration was done again by injecting 4 M MgCl_2_. Kinetic measurements consisted of at least five different concentrations ECL (1, 2, 10, 20 and 100 µL), while one of them was determined twice; additionally one blank was included. For every protein sample (**Gal-1** and **Gal-3**), *K*_D_s were determined twice. For the TTL without galactose units (**Gal-0**), binding was measured once at the highest possible lectin concentration (100 µM). Data were aligned and after additional subtraction of the blank measurement from each sensorgram (Figures S12–S14, Supporting Information File 1), analyzed on equilibrium binding by nonlinear curve fitting of the Langmuir binding isotherm (Figures S15 and S16, Supporting Information File 1).

## Supporting Information

Details on materials, protein design, construction of the expression plasmids, protein expression and purification, mass spectrometry data for the expressed proteins, general methods, synthetic protocols and analytical data (including ^1^H, ^13^C and ^19^F NMR spectra) for compounds **1** and **2**, reaction conditions for the ligation strategies, SDS PAGE and Western Blot lanes are provided as Supporting Information.

File 1 Additional data.
