# Supplementary material for: Orthogonal dual-modification of proteins for the engineering of multivalent protein scaffolds
Source: Beilstein J Org Chem. 2015 May 13;11:784–91. doi: 10.3762/bjoc.11.88 (PMC4464295; doi:10.3762/bjoc.11.88)
Supplement: File 1 — Additional data. [file Beilstein_J_Org_Chem-11-784-s001.pdf]

Supporting Information

for

Orthogonal dual-modification of proteins for the engineering of multivalent protein scaffolds

Michaela Mühlberg[‡,1,2], Michael G. Hoesl[‡,3], Christian Kuehne[4], Jens Dernedde[4], Nediljko Budisa*[,3] and Christian P. R. Hackenberger*[,§,1,5]

Address: [1]Forschungsinstitut für Molekulare Pharmakologie (FMP), Robert-Roessle-Str. 10, 13125 Berlin, Germany, [2]Freie Universität Berlin, Institut für Chemie und Biochemie, Takustr. 3, 14195 Berlin, Germany, [3]Technische Universität Berlin, AK Biokatalyse, Institut für Chemie, Müller-Breslau-Str. 10, 10623 Berlin, Germany, [4]Charité - Universitätsmedizin Berlin, Institut für Laboratoriumsmedizin, Klinische Chemie und Pathobiochemie, Augustenburger Platz 1, 13353 Berlin, Germany and [5]Humboldt Universität zu Berlin, Institut für Organische und Bioorganische Chemie, Institut für Chemie, Brook-Taylor-Str. 2, 12489 Berlin, Germany

Email: Christian P. R. Hackenberger - hackenbe@fmp-berlin.de, Nediljko Budisa - nediljko.budisa@tu-berlin.de

*Corresponding author

§Fax (C.P.R.H.): +49 (0)30 94793 188,

[‡]Authors have contributed equally.

Materials

Reagents and solvents, unless stated otherwise, were purchased from commercial suppliers and used without further purification. Dry solvents (benzene, DMSO, dichloromethane, acetonitrile) were purchased from ACROS ORGANICS. TBTA was synthesized according to the literature[1] and provided by members of the group. Azidohomoalanine (Aha) was synthesized as described elsewhere[2] or purchased from Chiralix (Nijmegen, The Netherlands). Enzymes for standard cloning procedures were from New England Biolabs (Frankfurt am Main, Germany).

Protein design

In standard protein expression, the *N*-terminal methionine is cleaved off by methionine amino peptidase (Map) when followed by small amino acids like glycine, alanine or serine. This process is called *N*-terminal methionine excision (NME). Thus, Ser2 is readily available for *N*-terminal oxime ligation. However, incorporation of Aha is known to hamper NME and leaves a mixture of Ser- and AhaSer N-terminus.[3] To avoid this mixture, we constructed a TTL mutant which contained an *N*-terminal His-tag followed by a TEV cleavage (cutting site Glu-Asn-Leu-Tyr-Phe-Gln↓Ser) site which should enable not only His-tag removal but also a homogenous protein preparation with an *N*-terminal serine. However, no conditions were found under which the tag could be successfully removed. The introduction of an additional three amino acid spacer sequence (Pro-Ala-Ser) between TEV cleavage site and protein was unproductive as well (data not shown). Therefore, we engineered an alternative variant with a *C*-terminal His-tag and an *N*-terminal Met-Ser-Gly-Ser sequence which successfully allowed us to modify the N-terminus thereby accepting certain Ser/Aha-Ser heterogeneity.

In the final expression construct, we conservatively removed Met83 by mutation to a Leu because we found TTL to contain an alternative open reading frame starting with Met83 which led to slightly heterogeneous protein preparations in the past.[4] This leads to a final amount of nine methionine residues plus the Met1.

Construction of expression plasmids

MS-TTL-TEV-H6 was ordered as a synthetic gene from Geneart (Regensburg, Germany) containing the following modifications from the published TTL sequence:[5] i) an SGS sequence was introduced at the N-terminus, ii) a TEV protease cleavage site was introduced between gene sequence and C-terminal His-tag, and iii) aspartate 221 was mutated to cysteine. The gene was introduced between the EcoRI and PstI sites of pQE80L. Subsequently, the mutations M83L and C221D were introduced by site-directed mutagenesis using a standard Quikchange PCR protocol to obtain the expression construct pQE80L_MS-TTL-TEV-H6(M83L).

DNA sequence

*gaattc*attaaagaggagaaattaagcATGAGCGGATCCCAAAAGGCTGTTGAAATTACATATAACGGCAAAACTTTA
AGAGGAATGATGCATTTGCCTGATGATGTTAAGGGTAAAGTGCCTATGGTAATAATGTTTCACGGTTTTACAGGCAAT
AAAGTAGAGTCTCACTTTATTTTTGTGAAGATGTCAAGAGCTTTAGAAAAAGTAGGTATTGGGAGTGTAAGGTTTGAC
TTTTATGGTTCTGGAGAAAGTGATGGGGACTTTAGTGAACTGACATTTAGCAGTGAATTGGAAGATGCAAGACAAATT
TTAAAGTTTGTGAAAGAGCAACCTACGACTGACCCTGAGAGAATAGGACTACTTGGTTTGAGTATGGGAGGAGCTATT
GCAGGGATTGTAGCAAGGGAATATAAAGATGAAATAAAGGCGTTGGTGCTATGGGCTCCAGCTTTTAATATGCCTGAG
CTTATAATGAACGAAAGTGTAAAGCAATACGGAGCTATTATGGAACAATTGGGCTTTGTAGACATAGGAGGACATAAA
CTGAGTAAAGATTTTGTTGAGGATATTTCAAAATTAAATATATTTGAGCTGTCAAAAGGATACGATAAAAAAGTGCTT
ATAGTTCATGGGACAAATGATGAAGCGGTTGAATATAAAGTTTCTGATAGAATCTTAAAAGAGGTTTATGGGGACAAC
GCTACAAGAGTGACAATCGAAAATGCAGACCATACTTTTAAGAGTTTAGAATGGGAGAAAAAGGCGATTGAGGAGTCA
GTAGAGTTTTTCAAAAAGGAATTGTTAAAGGGAGGATCCGAGAACCTGTACTTCCAATCCGCCcatcaccatcaccat
cactgataa*ctgcag*

Protein sequence.

```
  1 MSGSQKAVEITYNGKTLRGMMHLPDDVKGKVPMVIMFHGFTGNKVESHFIFVKMSRALEK 60
 61 VGIGSVRFDFYGSGESDGDFSELTFSSELEDARQILKFVKEQPTTDPERIGLLGLSMGGA 120
121 IAGIVAREYKDEIKALVLWAPAFNMPELIMNESVKQYGAIMEQLGFVDIGGHKLSKDFVE 180
181 DISKLNIFELSKGYDKKVLIVHGTNDEAVEYKVSDRILKEVYGDNATRVTIENADHTFKS 240
241 LEWEKKAIEESVEFFKKELLKGGSENLYFQSAHHHHHH                       279
```

Methionine positions are marked in red. In Ser-TTL[Aha], these positions are occupied by Aha. The *N*-terminal Aha is partly cleaved off (see Figure S1).

Protein expression and purification.

Ser-TTL-TEV-H6[Aha] was expressed using the methionine auxotrophic *E. coli* strain B834(DE3) which was transformed with pQE80L-MS-TTL-TEV-H6(M83L). Cells were grown at 30 °C in New Minimal Medium[6] containing of Met (40 µM) as natural substrate until depletion at OD_{600} between 0.6–0.8. Subsequently, 100 mg/L Aha was added to the medium and target protein expression was induced by addition of isopropyl β-D-1-thiogalactopyranoside (IPTG, 1 mM). Expression was performed for 4 h at 30 °C. Cells were harvested by centrifugation (4000 × g, 4 °C, 15 min), resuspended in sodium phosphate buffer (100 mM, pH 7.5) supplemented with 0.1% Triton X-100, 0.5 mg/mL lysozyme, and 1 mg/mL of DNase and RNase, and incubated for 30 min. Subsequently, cells were lysed by sonication. Lysate clearing was performed by centrifugation (15,000 rpm, 4 °C, 30 min). For purification, NaCl was added to the lysate in a final concentration of 500 mM. TTL was purified by Ni-NTA chromatography with a linear gradient of 0–500 mM imidazole. TTL containing fractions were pooled and dialyzed against sodium phosphate buffer (100 mM, pH 7.2, 100 mM NaCl). Protein concentration was determined by UV_{280} absorbance and using the ε_M from the software ProtParam provided by the Expasy Proteomics Server (www.expasy.ch/tools/#proteome).

Mass spectrometry of expressed proteins

MS analysis of full length proteins was performed on an LTQ-FT Ultra mass spectrometer (Thermo Scientific) coupled online to an Ultimate 3000 HPLC Instrument (Thermo Scientific). Desalting was carried out with Massprep online desalting cartridges (Waters). Briefly, proteins were loaded in 1% formic acid and eluted in a 5 min gradient from 6 to 95% acetonitrile, 1% formic acid. Spectra were acquired in full scan mode with a resolution of 200.000 at m/z 400 and afterwards deconvoluted with the software Promass (Thermo Scientific) using basic deconvolution default settings for a mass range of 25000 to 35000 Da.

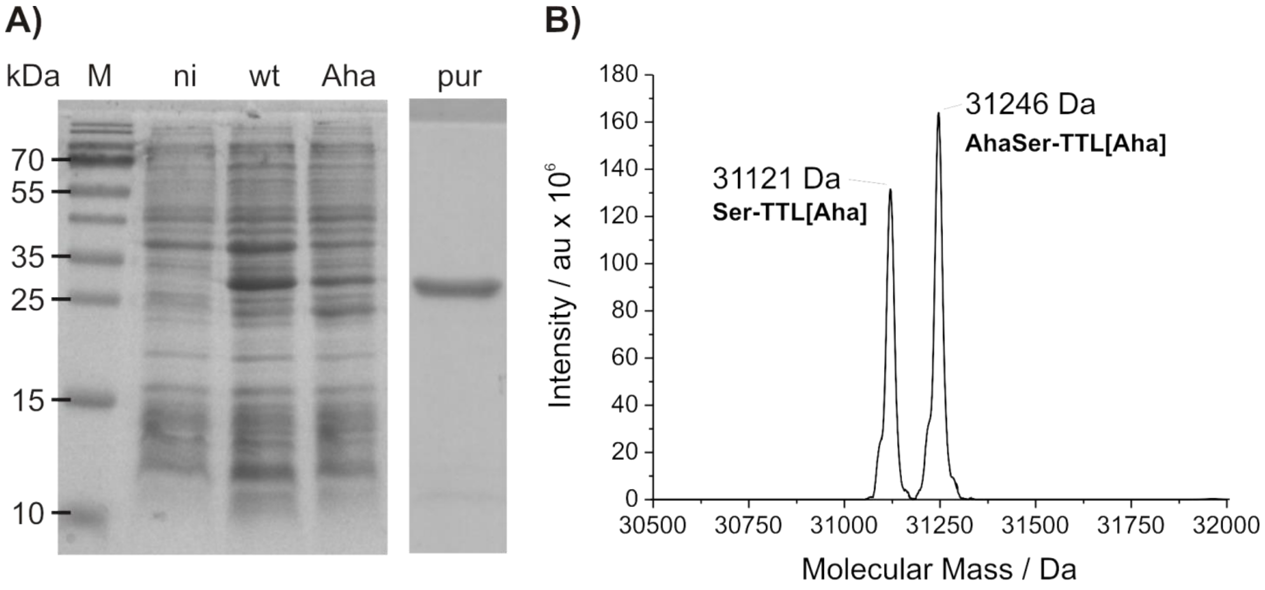

Figure S1: Expression, purification and mass analysis of Ser-TTL[Aha]. A) Coomassie stained 12% SDS gel with 0.15 OD_{600} of cell lysates per lane of *E. coli* B834(DE3){pQE80L_MS-TTL-TEV-H6(M83L)}. **M:** marker (PageRuler Prestained Protein Ladder); **ni**: non-induced sample; **wt**: Ser-TTL expression; **Aha**: (Aha)Ser-TTL[Aha] expression; **pur**: 5 µg of purified (Aha)Ser-TTL[Aha]. **B)** ESI-MS spectrum of (Aha)Ser-TTL[Aha]. The spectrum reveals complete incorporation of Aha. Two species are detected which can be assigned to Ser-TTL[Aha] with (theoretical mass: 31245 Da) and without (theoretical mass 31119 Da) *N*-terminal Aha. The amount of Ser-TTL[Aha] with N-terminal serine was determined to 45% by peak integration. We were able to purify 5 mg/L/OD_{600} of fully Aha labeled protein. With the standard construct of TTL we usually purify around 25 mg/L/OD_{600} of protein.

General methods

Thin-layer chromatography (TLC) was performed with precoated silica gel plates and visualized by UV light (λ = 254 nm) or $KMnO_4$ solution. The reaction mixtures were purified by column chromatography over silica gel (60−240 mesh). ^{1}H-NMR, ^{13}C-NMR and ^{19}F-NMR spectra were recorded on a Jeol ECX/400 or Bruker AVANCE 500 in $(CD_3)_2SO$, $CDCl_3$, CD_3OD or D_2O. The chemical shifts are reported in ppm relatively to the residual solvent peak. For TFA-quantifiaction, trifluoroethanol was added to NMR samples prior to ^{19}F-NMR analysis. High-resolution mass spectra (HRMS) were collected with an Agilent 6210 ToF LC/MS system (Agilent Technologies, Santa Clara, California, USA) using water and acetonitrile in a 1:1 mixture (with 0.1% TFA) as eluent at a flow rate of 0.2 mL/min. UV spectra of the proteins were measured on a JASCO V-630 UV-VIS-spectrometer. MALDI measurements were performed with a MALDI-TOF-TOF instrument (AB SCIEX TOF/TOF 5800; Applied Biosystems, Framingham, MA, USA) equipped with an Nd:YAG laser. A solution of 7.6 mg of 2,5-dihydroxyacetophenone (DHAP) in ethanol (375 µL) was mixed with an 18 mg/mL aqueous solution of diammonium hydrogen citrate (125 mL) and used as matrix. Samples were prepared by mixing 1 µL of the protein solution with 1 µL matrix solution. From the resulting mixture, 1 µL was applied to the sample plate and samples were dried in air at room temperature. Analysis was performed in the in the linear positive ion mode. For each spectrum 10,000 consecutive laser shots were accumulated. Mass spectra were externally calibrated with lysozyme and analyzed with the Data Explorer Software (Applied Biosystems). SPR measurements were carried out at 25 °C on a Biacore X instrument (GE Healthcare, Freiburg, Germany).

Synthesis protocols

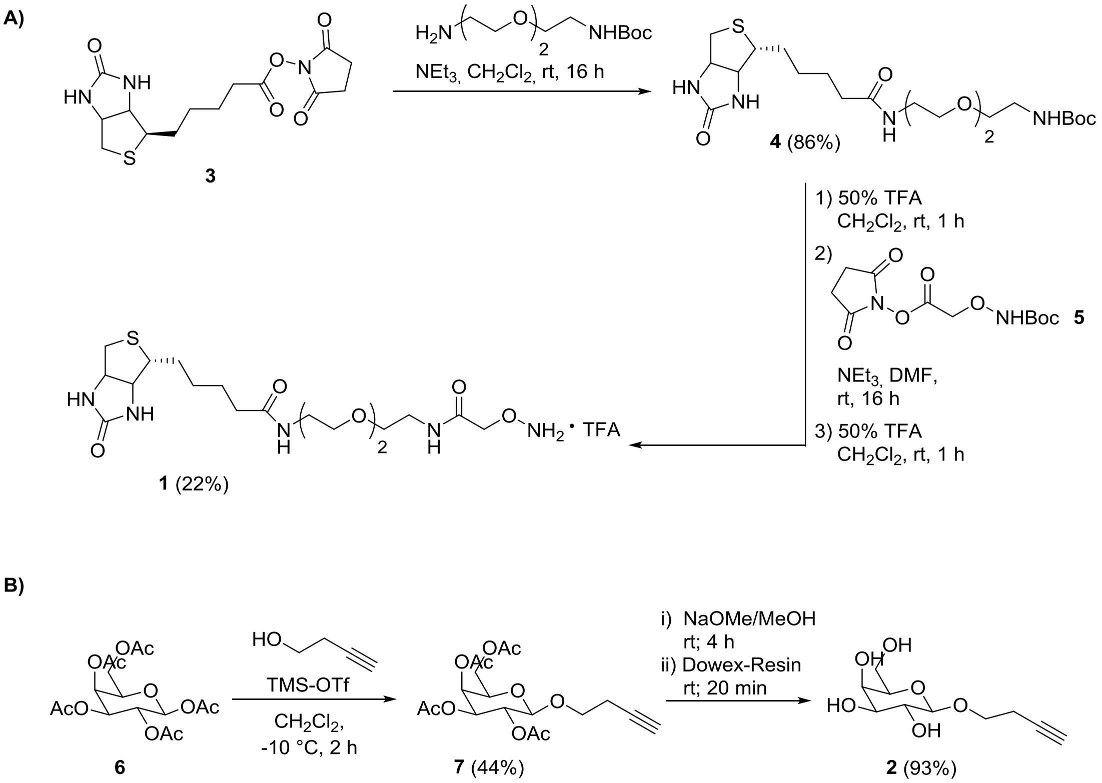

Scheme S1: Synthesis of **A)** biotin hydroxylamine **1**, **B)** *β*-butynyl galactose **2**.

(+)-Biotin *N*-hydroxysuccinimide ester (3). To a solution of (+)-biotin (0.82 mmol, 200 mg) and *N*-hydroxysuccinimide (0.89 mmol, 102 mg) was added 3-(ethyliminomethylidenamino)-*N*,*N*-dimethylpropan-1-amine hydrochloride (0.96 mmol, 184 mg). The reaction mixture was stirred overnight at room temperature and concentrated to give a white solid. The crude solid was mixed with isopropanol, heated up to 70 °C and cooled down. The pure product was filtered off as white powder in 91% yield (0.75 mmol, 255 mg): ^{1}H-NMR ($(CD_3)_2SO$, 400 MHz): δ(ppm) = 6.43 (s, 1H), 6.37 (s, 1H), 4.35-4.27 (m, 1H), 4.19-4.08 (m, 1H), 3.10 (dd, *J* = 11.8, 7.2 Hz, 1H), 2.92-2.78 (m, 5H), 2.67 (t, *J* = 7.4 Hz, 2H), 2.58 (d, *J* = 12.5 Hz, 1H), 1.71-1.34 (m, 6H); ^{13}C-NMR ($(CD_3)_2SO$, 100 MHz): δ(ppm) = 170.3, 169.0, 162.7, 61.0, 59.2, 58.9, 55.3, 30.0, 27.8, 27.6, 25.5, 24.3. The analytical data is in accordance with the literature.[7]

***tert*-Butyl-(2-(2-(2-(5-((3a*S*,4*S*,6a*R*)-2-oxohexahydro-1*H*-thieno[3,4-*d*]imidazol-4-yl)pentanamido)ethoxy)-ethoxy)ethyl)carbamate (4).** Biotin derivative **3** (0.29 mmol, 100 mg), Boc-1-amino-3,6-dioxa-8-octanamine (0.44 mmol, 109 mg) and triethylamine (0.59 mmol, 81.0 µL) were dissolved in dry DMF (5 mL). The reaction mixture was stirred overnight at room temperature. DMF was removed, the residue taken up with CH_2Cl_2 and washed with water. The organic layer was dried with $MgSO_4$ and concentrated to yield the crude product. Column chromatography (CH_2Cl_2 + MeOH (slowly increasing from 0% to 7.5%)) yielded the desired product **4** in 86% (0.25 mmol, 118 mg) as a white sticky solid: R_f(CH_2Cl_2:MeOH; 9:1) 0.4; ^{1}H-NMR ($CDCl_3$, 400 MHz): δ(ppm) = 6.74 (s, 1H), 5.94 (s, 1H), 4.53-4.39 (m, 1H), 4.34-4.20 (m, 1H), 3.66-3.47 (m, 8H), 3.40 (dd, *J* = 10.1, 5.1 Hz, 2H), 3.33-3.22 (m, 2H), 3.11 (dd, *J* = 11.8, 7.1 Hz, 1H), 2.86 (dd, *J* = 12.8, 4.8 Hz, 1H), 2.71 (d, *J* = 12.8 Hz, 1H), 2.20 (t, *J* = 7.5 Hz, 2H), 1.79-1.54 (m, 4H), 1.49-1.32 (m, 10H), 1.27-1.17 (m, 1H); ^{13}C-NMR ($CDCl_3$, 100 MHz): δ(ppm) = 173.5, 164.4, 156.1, 79.4, 70.1, 61.9, 60.3, 55.8, 40.6, 40.4, 39.2, 36.1, 28.5, 28.4, 28.2, 25.7. The analytical data is in accordance with the literature.[8]

2,5-Dioxopyrrolidin-1-yl 2-(((*tert*-butoxycarbonyl)amino)oxy)acetate (5). 2-(((*tert*-Butoxycarbonyl)amino)-oxy)acetic acid (1.50 mmol, 287 mg) and *N*-hydroxysuccinimide (1.65 mmol, 190 mg) were dissolved in dry dimethylformamide (DMF). 3-(Ethyliminomethyleneamino)-*N*,*N*-dimethylpropan-1-amine hydrochloride (EDC-HCl) (1.80 mmol, 227 mg) was added and the reaction was stirred overnight at room temperature. The mixture was diluted with water and extracted with ethylacetate. The organic layer was dried and concentrated to yield the product as a yellow liquid with some DMF impurities (≈59%, 255 mg). The product was applied in the next reaction step without further purification.

***N*-(2-(2-(2-(2-(Aminooxy)acetamido)ethoxy)ethoxy)ethyl)-5-((3a*S*,4*S*,6a*R*)-2-oxohexahydro-1*H*-thieno[3,4-*d*]imidazol-4-yl)pentanamide (1).** Biotin derivative **4** (0.24 mmol, 115 mg) was dissolved in CH_2Cl_2/TFA (4:1) and stirred for 1 h. The mixture was concentrated and dried under vacuum. The residue was redissolved in dry DMF with compound **5** (0.48 mmol, 1.40 mg) and triethylamine (0.58 mmol, 58.9 mg) and stirred at room temperature overnight. Concentration and purification by column chromatography (CH_2Cl_2 + MeOH (slowly increasing from 0% to 7%); R_f(CH_2Cl_2:MeOH; 9:1) 0.3) yielded the Boc-protected product. The compound was then redissolved in CH_2Cl_2/TFA (4:1) and stirred for 1 h. The mixture was concentrated and dried under vaccum to yield the desired product **1** as TFA salt (1:1.16) in 22 % (53.0 µmol, 30.7 mg) as a white sticky solid: ^{1}H-NMR (D_2O, 400 MHz): δ(ppm) = 4.73-4.59 (m, 3H), 4.45 (dd, *J* = 7.6, 4.6 Hz, 1H), 3.76-3.60 (m, 8H), 3.50 (t, *J* = 5.1 Hz, 2H), 3.46-3.31 (m, 3H), 3.02 (dd, *J* = 13.1, 4.8 Hz, 1H), 2.80 (d, *J* = 13.0 Hz, 1H), 2.30 (t, *J* = 7.1 Hz, 2H), 1.83-1.36 (m, 6H); ^{13}C-NMR (D_2O, 100 MHz): δ(ppm) = 176.98, 168.84, 165.35, 163.15, 162.68, 118.29, 114.43, 71.77, 69.44, 68.89, 68.63, 62.10, 60.27, 55.37, 39.69, 38.85, 38.70, 35.46, 27.85, 27.69, 25.13; ^{19}F-NMR (D_2O, 376 MHz): δ(ppm) = –75.5; HRMS: (ESI-ToF): m/z (calculated): $[M+H]^+$ = 448.2224 ($C_{18}H_{34}N_5O_6S^+$), m/z (experimental): $[M+H]^+$ = 448.2224.

2,3,4,6-Tetra-*O*-acetyl-1-*O*-but-3-ynyl-α-galactopyranoside (7). A solution of per-acetylated β-galactose **6** (0.76 mmol, 300 mg) and 3-butyn-1-ol (1.54 mmol, 120 µl) in dry CH_2Cl_2 was cooled down to −10 °C. TMS-OTf (3.84 mmol, 0.70 ml) was added dropwise. The reaction mixture was stirred for 2 h and poured into aq. sat. $NaHCO_3$-solution. Extraction with EtOAc (3 times), drying with $MgSO_4$ and concentration yielded the crude product. Column chromatography (EtOAc:cyclohexane; 1:4 → 1:2) gave the desired product **7** as a viscous oil in 44% yield (0.34 mmol, 136.7 mg): R_f(EtOAc:cyclohexane; 1:1) 0.6; ^{1}H-NMR ($CDCl_3$, 400 MHz): δ(ppm) = 5.39 (d, *J* = 3.3 Hz, 1H), 5.21 (dd, *J* = 10.4, 7.9 Hz, 1H), 5.01 (dd, *J* = 10.4, 3.3 Hz, 1H), 4.53 (d, *J* = 7.9 Hz, 1H), 4.23-4.05 (m, 2H), 3.94 (ddd, *J* = 20.2, 11.8, 6.7 Hz, 2H), 3.67 (dt, *J* = 9.7, 7.2 Hz, 1H), 2.48 (dt, *J* = 6.9, 2.6 Hz, 2H), 2.15 (s, 3H), 2.06 (s, 3H), 2.05 (s, 3H), 1.98 (s, 3H), 1.96 (dt, *J* = 2.6 Hz, 1H); ^{13}C-NMR ($CDCl_3$, 100 MHz): δ(ppm) = 170.3, 170.2, 170.1, 169.4, 101.3, 80.6, 70.8, 70.7, 69.6, 68.6, 67.9, 67.0, 61.3, 20.8, 20.6, 20.6, 20.5, 19.8. The analytical data is in accordance with the literature.[9]

1-*O*-But-3-ynyl-α-galactopyranoside (2). To a solution of **7** (0.42 mmol, 170 mg) in 10 ml methanol was added NaOMe (32% in MeOH, 0.40 ml) and the reaction was stirred for 6 h at room temperature. Dowex-Exchange-Resin (3 g) was added and stirring proceeded for 50 min. Filtration and concentration afforded the desired product **2** as a white solid in 93% yield (0.39 mmol, 91.1 mg): ^{1}H-NMR (CD_3OD, 400 MHz): δ(ppm) = 4.25 (d, *J* = 7.4 Hz, 1H), 3.96 (dt, *J* = 9.7, 7.3 Hz, 1H), 3.82 (dd, *J* = 3.2, 1.1 Hz, 1H), 3.80-3.65 (m, 3H), 3.54-3.48 (m, 2H), 3.46 (dd, *J* = 9.7, 3.2 Hz, 1H), 2.51 (td, *J* = 7.3, 2.7 Hz, 2H), 2.26 (t, *J* = 2.7 Hz, 1H); ^{13}C-NMR (CD_3OD, 100 MHz): δ(ppm) = 105.0, 81.8, 76.7, 74.9, 72.4, 70.6, 70.3, 69.0, 62.5, 20.6; HRMS: (ESI-ToF): m/z (calculated): $[M+Na]^+$ = 255.0839 ($C_{10}H_{16}O_6Na^+$), m/z (experimental): $[M+Na]^+$ = 255.0835.

NMR spectra of final compounds

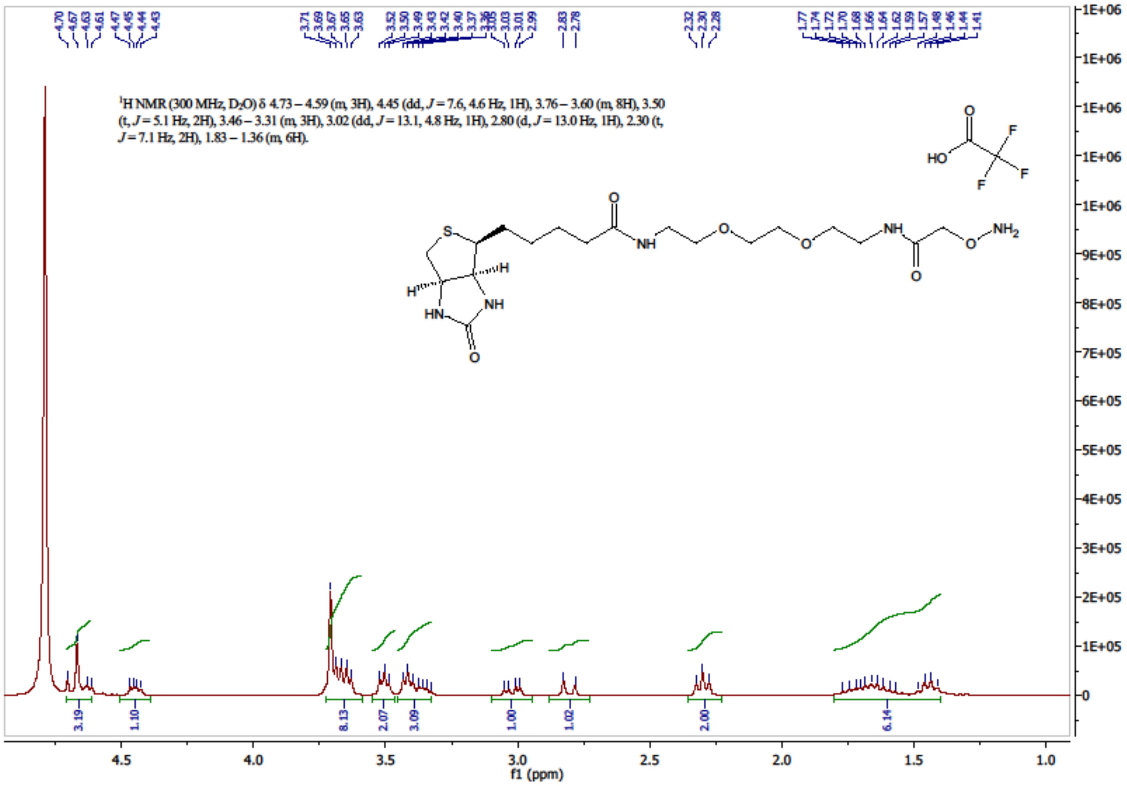

Figure S2: ^{1}H-NMR (D_2O, 400 MHz) of compound **1**.

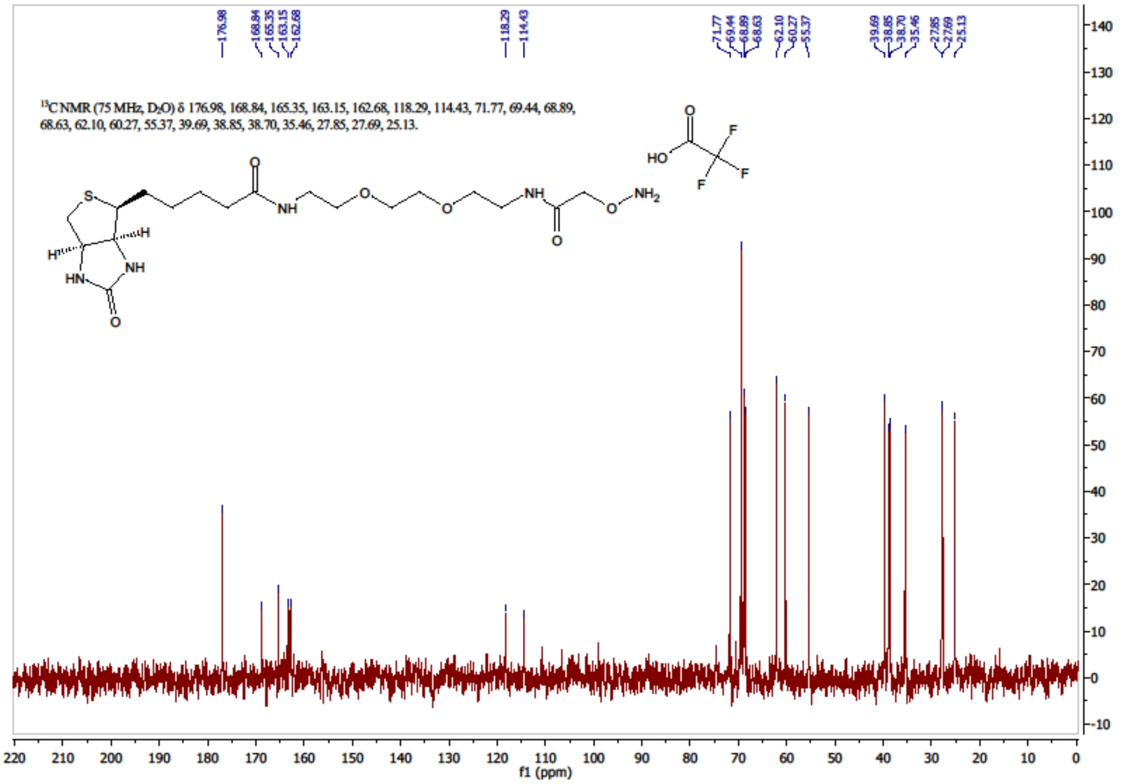

Figure S3: ^{13}C-NMR (D_2O, 100 MHz) of compound **1**.

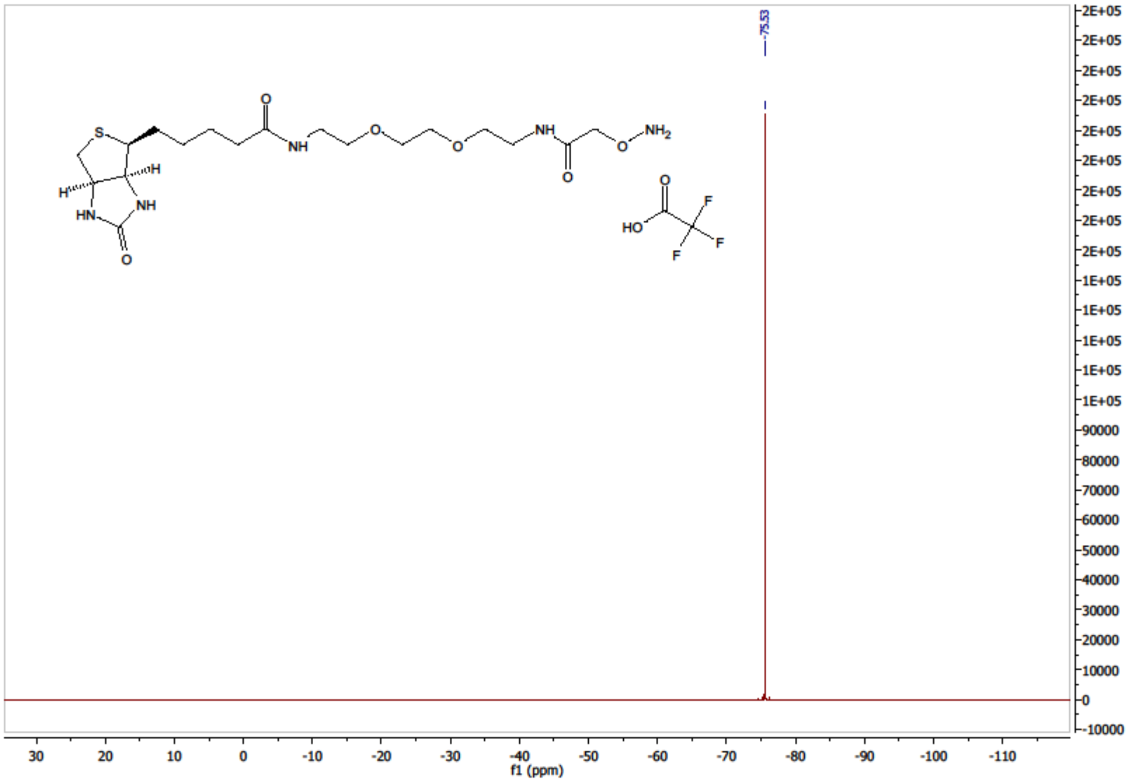

Figure S4: ^{19}F-NMR (D_2O, 376 MHz) of compound **1**.

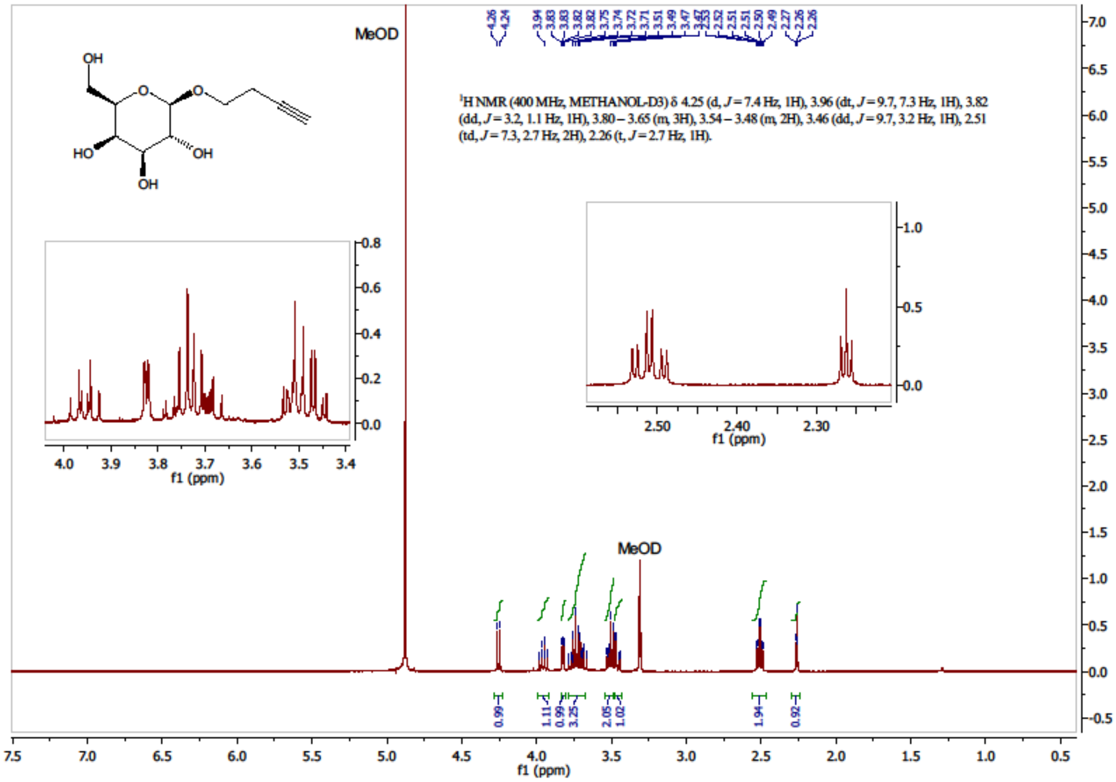

Figure S5: ^{1}H-NMR (CD_3OD, 400 MHz) of compound **2**.

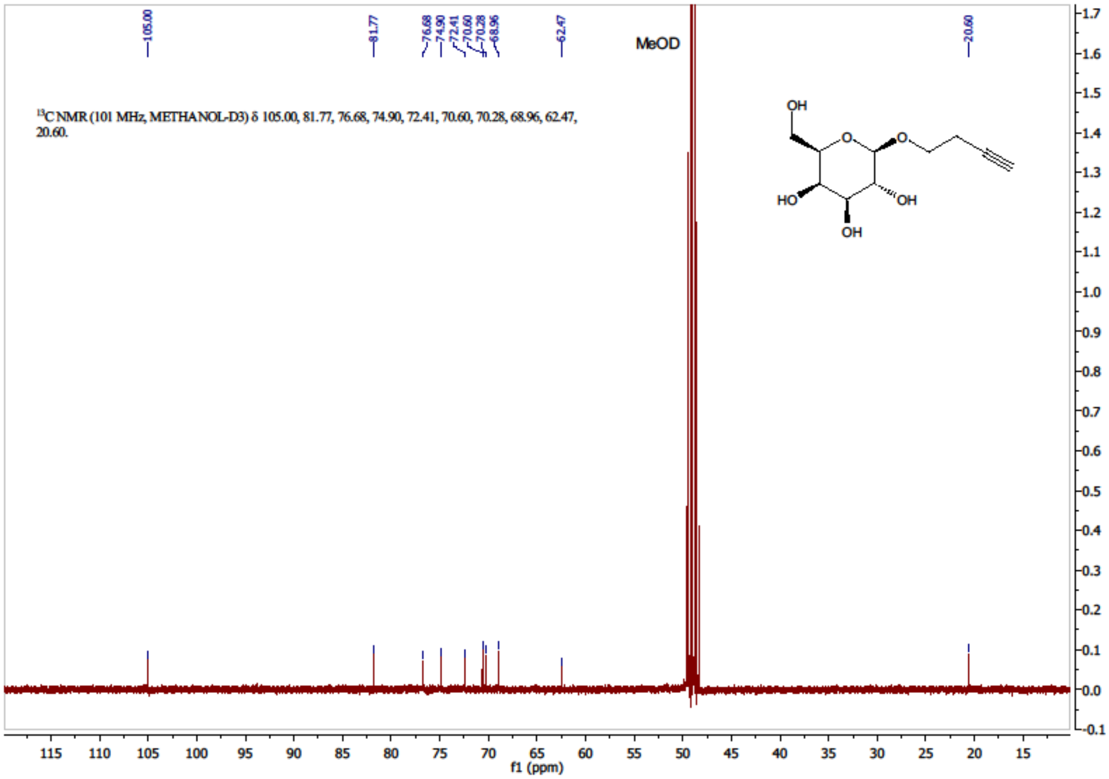

Figure S6: ^{13}C-NMR (CD_3OD, 100 MHz) of compound **2**.

Bio-orthogonal protein labeling

Table S1: Conditions for oxime ligation on TTL†

entry	reagent 1	pH	catalyst (10 eq.)	conversion*
**1**	30 equiv	7.4	*p*-anisidine	10%
**2**	50 equiv	5.0	–	50%
**3**	30 equiv	5.0	–	30%
**4**	30 equiv	4.5	–	60%
**5**	30 equiv	4.0	–	65%
**6**	30 equiv	3.5	–	80%
**7**	30 equiv	3.0	–	100%
**8**	20 equiv	3.0	–	100%
**9**	10 equiv	3.0	–	80%
**10**	5 equiv	3.0	–	55%

† 50 µM, 15 °C, overnight. * Estimated by MALDI-MS in comparison to unreacted AhaSer-TTL[Aha].

Table S2: Conditions for CuAAC on biotinylated TTL†

entry	c [µM] (protein)	$CuSO_4$ (to alkyne)	product*
**1**	10	5 mol %	3-5 clicked sugars
**2**	10	10 mol %	3-4 clicked sugars (**Gal-3**)
**3**	10	30 mol %	1-2 clicked sugars (**Gal-1**)
**4**	10	50 mol %	1-2 clicked sugars

† 5 equiv THPTA to Cu^{2+}, 8 mM aminoguanidine, 100 equiv of reagent **2** per azide, 50 eq. sodium ascorbate/Cu^{2+}, 15 °C, overnight.
* Ratios judged by MALDI-MS in comparison to biotinylated TTL (Table S1, entry 8).

MALDI-ToF spectra

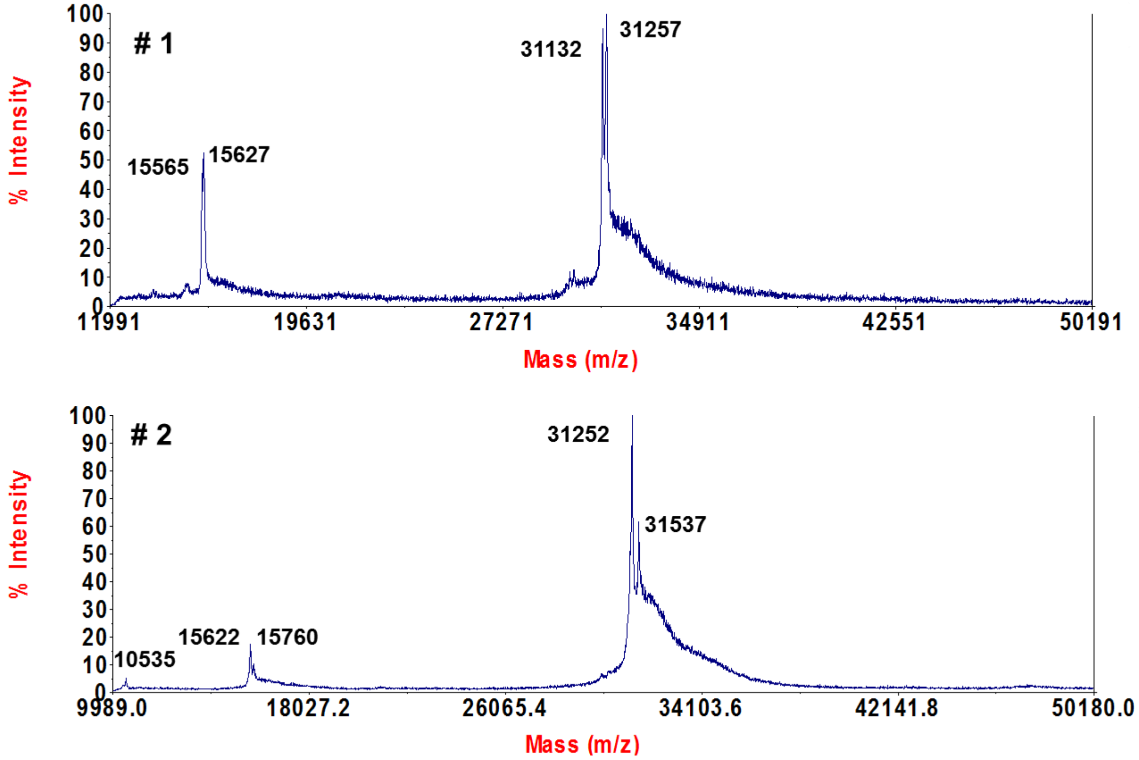

Figure S7: MALDI-ToF spectra (see Table S3, entries 1 and 2).

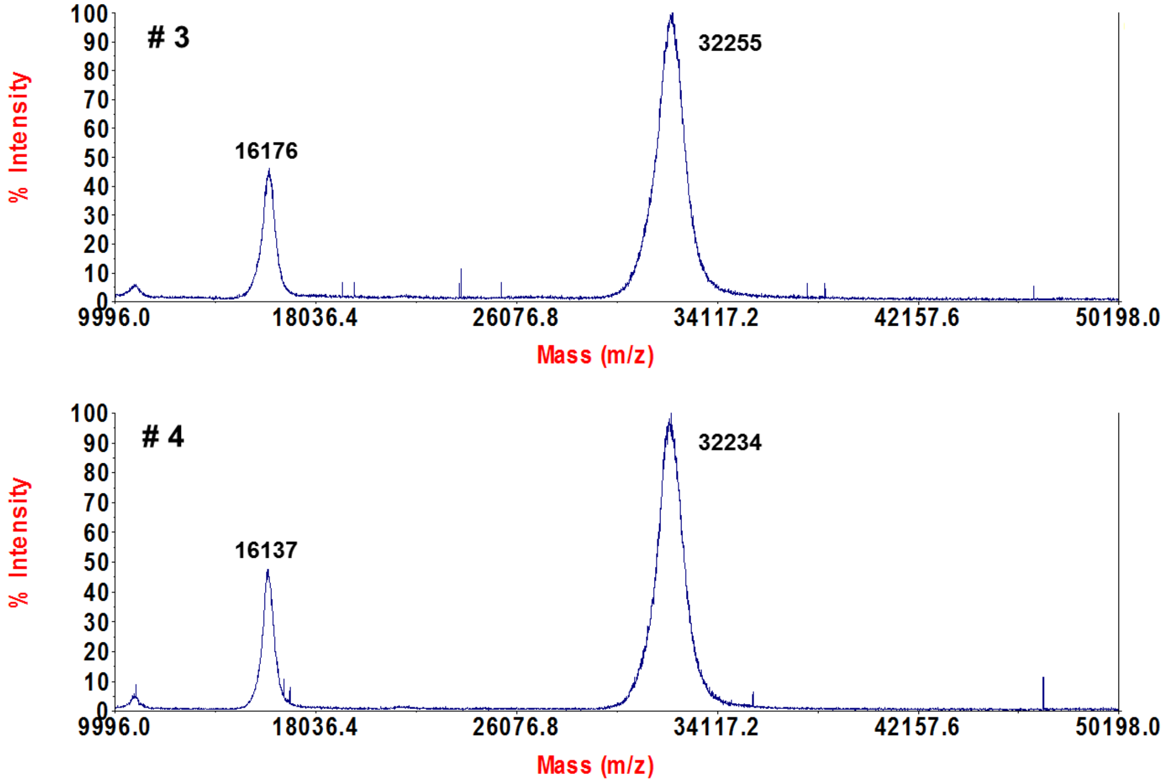

Figure S8: MALDI-ToF spectra (see Table S3, entries 3 and 4).

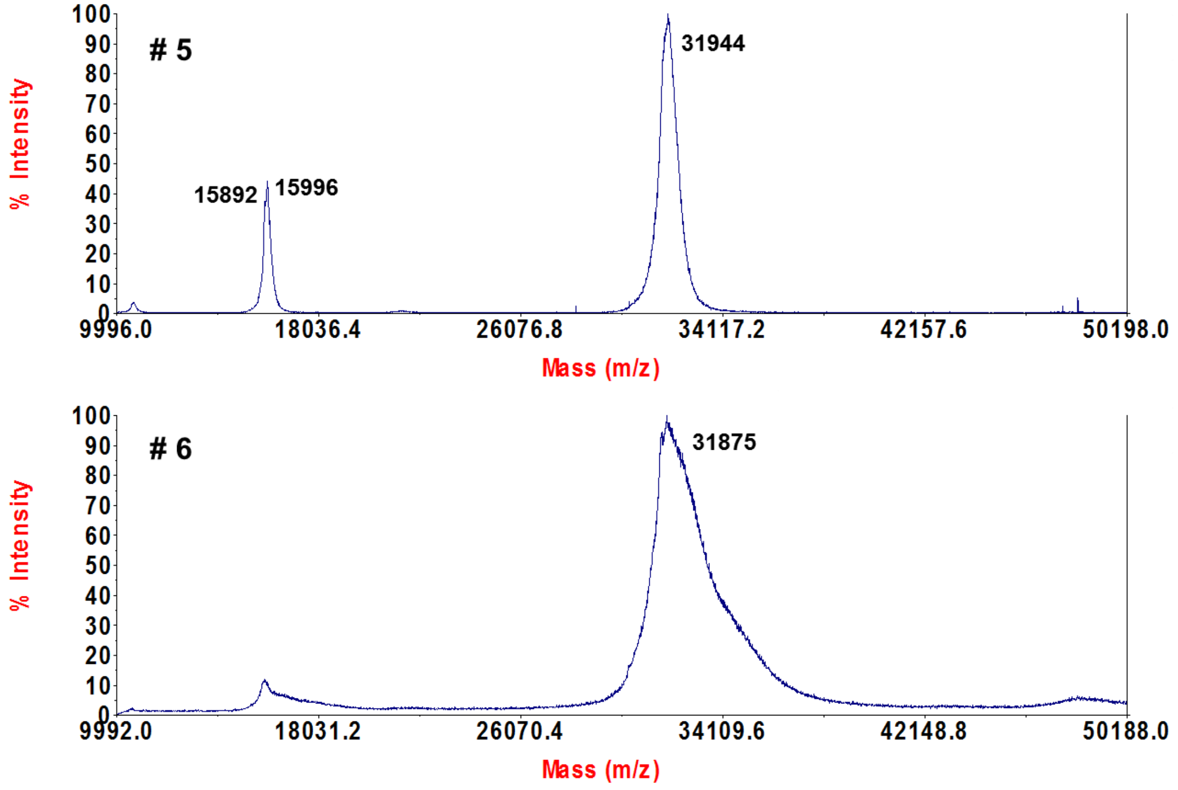

Figure S9: MALDI-ToF spectra (see Table S3, entries 5 and 6).

Table S3: Experimental MALDI-ToF data for protein samples.

entry	protein sample	$[M+H]^+$ (experimental, average)
# 1	TTL lipase	31132 + (31257) Da
# 2	Biotin TTL Lipase (Table 1, entry 3) = **Gal-0**	(31252) + 31537 Da
# 3	Biotin (Gal-)TTL Lipase (Table 2, entry 1)	peak max.: ≈32255
# 4	Biotin (Gal-)TTL Lipase (Table 2, entry 2) = **Gal-3**	peak max.: ≈32234
# 5	Biotin (Gal-)TTL Lipase (Table 2, entry 3) = **Gal-1**	peak max.: ≈31944
# 6	Biotin (Gal-)TTL Lipase (Table 2, entry 4)	peak max.: ≈31875

Table S4: Theoretical MALDI-ToF data for protein samples.

protein sample	$[M+H]^+$ (theoretical, average)
TTL lipase	31119 + (31245) Da
Biotin TTL Lipase	(31245) + 31517 Da
Biotin TTL Lipase + 1 Gal	(31477) + 31749 Da
Biotin TTL Lipase + 2 Gal	(31709) + 31981 Da
Biotin TTL Lipase + 3 Gal	(31942) + 32214 Da
Biotin TTL Lipase + 4 Gal	(32174) + 32446 Da
Biotin TTL Lipase + 5 Gal	(32406) + 32678 Da

Slight mass differences are due to a limited resolution of MALDI-MS measurements of proteins.

Protein digest

The protein digest and MS/MS analysis of protein mixture **Gal-3** was performed according to the literature.[10] For the results see Table S5.

Table S5: Mascot search results from trypsin digested **Gal-3**.

Start	End	Observed	Mr(expt)	Mr(calc)	ppm	Miss	Sequence
1	18	737	2.209	2.209	7	2	-.AhaSGSQKAVEITYNGKTLR.G Click Produkt MM (A) (Ions score 27)
7	15	498	994	994	**1**	0	K.AVEITYNGK.T (Ions score 34)
7	15	498	994	994	6	0	K.AVEITYNGK.T (Ions score 38)
7	15	498	994	994	6	0	K.AVEITYNGK.T (Ions score 35)
7	15	498	994	994	7	0	K.AVEITYNGK.T (Ions score 38)
7	18	456	1.364	1.364	8	1	K.AVEITYNGKTLR.G (Ions score 42)
7	28	827	2.477	2.477	5	2	K.AVEITYNGKTLRGAhaAhaHLPDDVK.G 2 Azidohomoalanin (A) (Ions score 3)
16	28	484	1.450	1.450	8	1	K.TLRGAhaAhaHLPDDVK.G 2 Azidohomoalanin red. (A) (Ions score 17)
16	28	493	1.476	1.476	8	1	K.TLRGAhaAhaHLPDDVK.G Azidohomoalanin (A); Azidohomoalanin red. (A) (Ions score 28)
16	28	502	1.502	1.502	7	1	K.TLRGAhaAhaHLPDDVK.G 2 Azidohomoalanin (A) (Ions score 21)
19	28	554	1.106	1.106	5	0	R.GAhaAhaHLPDDVK.G Azidohomoalanin (A); Azidohomoalanin red. (A) (Ions score 24)
19	28	370	1.106	1.106	7	0	R.GAhaAhaHLPDDVK.G Azidohomoalanin (A); Azidohomoalanin red. (A) (Ions score 18)
19	28	370	1.106	1.106	9	0	R.GAhaAhaHLPDDVK.G Azidohomoalanin (A); Azidohomoalanin red. (A) (Ions score 23)
19	28	378	1.132	1.132	3	0	R.GAhaAhaHLPDDVK.G 2 Azidohomoalanin (A) (Ions score 15)
19	28	378	1.132	1.132	3	0	R.GAhaAhaHLPDDVK.G 2 Azidohomoalanin (A) (Ions score 24)
19	28	378	1.132	1.132	6	0	R.GAhaAhaHLPDDVK.G 2 Azidohomoalanin (A) (Ions score 15)
19	28	378	1.132	1.132	7	0	R.GAhaAhaHLPDDVK.G 2 Azidohomoalanin (A) (Ions score 24)
19	28	378	1.132	1.132	9	0	R.GAhaAhaHLPDDVK.G 2 Azidohomoalanin (A) (Ions score 21)
19	30	431	1.291	1.291	8	1	R.GAhaAhaHLPDDVKGK.V Azidohomoalanin (A); Azidohomoalanin red. (A) (Ions score 19)
19	30	440	1.317	1.317	7	1	R.GAhaAhaHLPDDVKGK.V 2 Azidohomoalanin (A) (Ions score 47)
19	30	440	1.317	1.317	7	1	R.GAhaAhaHLPDDVKGK.V 2 Azidohomoalanin (A) (Ions score 56)
19	30	440	1.317	1.317	8	1	R.GAhaAhaHLPDDVKGK.V 2 Azidohomoalanin (A) (Ions score 19)
29	44	576	1.726	1.726	4	1	K.GKVPAhaVIAhaFHGFTGNK.V Azidohomoalanin (A); Azidohomoalanin red. (A) (Ions score 16)
29	44	585	1.752	1.752	6	1	K.GKVPAhaVIAhaFHGFTGNK.V 2 Azidohomoalanin (A) (Ions score 32)
29	53	947	2.839	2.839	4	2	K.GKVPAhaVIAhaFHGFTGNKVESHFIFVK.A 2 Azidohomoalanin (A) (Ions score 17)
31	44	506	1.515	1.515	5	0	K.VPAhaVIAhaFHGFTGNK.V 2 Azidohomoalanin red. (A) (Ions score 12)
31	44	515	1.541	1.541	2	0	K.VPAhaVIAhaFHGFTGNK.V Azidohomoalanin (A); Azidohomoalanin red. (A) (Ions score 12)
31	44	515	1.541	1.541	7	0	K.VPAhaVIAhaFHGFTGNK.V Azidohomoalanin (A); Azidohomoalanin red. (A) (Ions score 28)

31	44	515	1.541	1.541	9	0	K.VPAhaVIAhaFHGFTGNK.V Azidohomoalanin (A); Azidohomoalanin red. (A) (Ions score 22)
31	44	523	1.567	1.567	6	0	K.VPAhaVIAhaFHGFTGNK.V 2 Azidohomoalanin (A) (Ions score 20)
31	44	523	1.567	1.567	7	0	K.VPAhaVIAhaFHGFTGNK.V 2 Azidohomoalanin (A) (Ions score 28)
31	53	885	2.653	2.653	7	1	K.VPAhaVIAhaFHGFTGNKVESHFIFVK.A 2 Azidohomoalanin (A) (Ions score 27)
31	53	885	2.653	2.653	7	1	K.VPAhaVIAhaFHGFTGNKVESHFIFVK.A 2 Azidohomoalanin (A) (Ions score 26)
45	53	369	1.105	1.105	7	0	K.VESHFIFVK.Aha (Ions score 21)
45	53	369	1.105	1.105	10	0	K.VESHFIFVK.Aha (Ions score 30)
45	56	492	1.474	1.474	7	1	K.VESHFIFVKAhaSR.A Azidohomoalanin (A) (Ions score 23)
57	67	377	1.128	1.128	9	1	R.ALEKVGIGSVR.F (Ions score 24)
68	93	1.454	2.906	2.906	7	0	R.FDFYGSGESDGDFSELTFSSELEDAR.Q (Ions score 78)
68	93	970	2.906	2.906	8	0	R.FDFYGSGESDGDFSELTFSSELEDAR.Q (Ions score 31)
68	97	1.131	3.389	3.389	7	1	R.FDFYGSGESDGDFSELTFSSELEDARQILK.F (Ions score 43)
94	100	438	875	875	7	1	R.QILKFVK.E (Ions score 35)
94	109	644	1.928	1.928	9	2	R.QILKFVKEQPTTDPER.I (Ions score 32)
98	109	483	1.446	1.446	7	1	K.FVKEQPTTDPER.I (Ions score 27)
98	109	483	1.446	1.446	8	1	K.FVKEQPTTDPER.I (Ions score 2)
98	109	483	1.446	1.446	8	1	K.FVKEQPTTDPER.I (Ions score 34)
98	109	483	1.446	1.446	8	1	K.FVKEQPTTDPER.I (Ions score 18)
98	109	483	1.446	1.446	8	1	K.FVKEQPTTDPER.I (Ions score 4)
98	127	1.031	3.091	3.091	7	2	K.FVKEQPTTDPERIGLLGLSAhaGGAIAGIVAR.E Azidohomoalanin (A) (Ions score 41)
101	109	537	1.071	1.071	7	0	K.EQPTTDPER.I (Ions score 17)
101	127	898	2.690	2.690	8	1	K.EQPTTDPERIGLLGLSAhaGGAIAGIVAR.E Azidohomoalanin red. (A) (Ions score 24)
101	127	907	2.716	2.716	7	1	K.EQPTTDPERIGLLGLSAhaGGAIAGIVAR.E Azidohomoalanin (A) (Ions score 22)
101	127	907	2.716	2.716	8	1	K.EQPTTDPERIGLLGLSAhaGGAIAGIVAR.E Azidohomoalanin (A) (Ions score 37)
101	127	984	2.949	2.949	6	1	K.EQPTTDPERIGLLGLSAhaGGAIAGIVAR.E Click Produkt MM (A) (Ions score 13)
110	127	547	1.637	1.637	6	0	R.IGLLGLSAhaGGAIAGIVAR.E Azidohomoalanin red. (A) (Ions score 51)
110	127	833	1.663	1.663	5	0	R.IGLLGLSAhaGGAIAGIVAR.E Azidohomoalanin (A) (Ions score 114)
110	127	833	1.663	1.663	5	0	R.IGLLGLSAhaGGAIAGIVAR.E Azidohomoalanin (A) (Ions score 57)
110	127	833	1.663	1.663	5	0	R.IGLLGLSAhaGGAIAGIVAR.E Azidohomoalanin (A) (Ions score 87)
110	127	833	1.663	1.663	6	0	R.IGLLGLSAhaGGAIAGIVAR.E Azidohomoalanin (A) (Ions score 108)

110	127	833	1.663	1.663	7	0	R.IGLLGLSAhaGGAIAGIVAR.E Azidohomoalanin (A) (Ions score 109)
110	127	555	1.663	1.663	7	0	R.IGLLGLSAhaGGAIAGIVAR.E Azidohomoalanin (A) (Ions score 32)
110	127	833	1.663	1.663	8	0	R.IGLLGLSAhaGGAIAGIVAR.E Azidohomoalanin (A) (Ions score 81)
110	127	833	1.663	1.663	9	0	R.IGLLGLSAhaGGAIAGIVAR.E Azidohomoalanin (A) (Ions score 91)
110	127	633	1.895	1.895	7	0	R.IGLLGLSAhaGGAIAGIVAR.E Click Produkt MM (A) (Ions score 23)
110	130	695	2.083	2.083	7	1	R.IGLLGLSAhaGGAIAGIVAREYK.D Azidohomoalanin (A) (Ions score 31)
110	134	839	2.513	2.513	-8	2	R.IGLLGLSAhaGGAIAGIVAREYKDEIK.A (Ions score 11)
110	134	857	2.568	2.568	7	2	R.IGLLGLSAhaGGAIAGIVAREYKDEIK.A Azidohomoalanin (A) (Ions score 17)
128	134	463	923	923	6	1	R.EYKDEIK.A (Ions score 30)
135	156	1.163	2.323	2.323	-3	0	K.ALVLWAPAFNMetAPELIMetNESVK.Q (Ions score 29)
157	174	653	1.957	1.957	4	0	K.QYGAIAhaEQLGFVDIGGHK.L Azidohomoalanin (A) (Ions score 46)
157	174	653	1.957	1.957	5	0	K.QYGAIAhaEQLGFVDIGGHK.L Azidohomoalanin (A) (Ions score 37)
157	174	653	1.957	1.957	6	0	K.QYGAIAhaEQLGFVDIGGHK.L Azidohomoalanin (A) (Ions score 26)
157	174	653	1.957	1.957	6	0	K.QYGAIAhaEQLGFVDIGGHK.L Azidohomoalanin (A) (Ions score 12)
157	174	653	1.957	1.957	8	0	K.QYGAIAhaEQLGFVDIGGHK.L Azidohomoalanin (A) (Ions score 15)
157	174	653	1.957	1.957	8	0	K.QYGAIAhaEQLGFVDIGGHK.L Azidohomoalanin (A) (Ions score 23)
157	174	653	1.957	1.957	9	0	K.QYGAIAhaEQLGFVDIGGHK.L Azidohomoalanin (A) (Ions score 42)
157	174	653	1.957	1.957	9	0	K.QYGAIAhaEQLGFVDIGGHK.L Azidohomoalanin (A) (Ions score 26)
157	177	754	2.259	2.259	-3	1	K.QYGAIAhaEQLGFVDIGGHKLSK.D Azidohomoalanin red. (A) (Ions score 10)
157	177	754	2.259	2.259	6	1	K.QYGAIAhaEQLGFVDIGGHKLSK.D Azidohomoalanin red. (A) (Ions score 24)
157	177	763	2.285	2.285	6	1	K.QYGAIAhaEQLGFVDIGGHKLSK.D Azidohomoalanin (A) (Ions score 47)
175	185	641	1.280	1.280	6	1	K.LSKDFVEDISK.L (Ions score 10)
175	185	428	1.280	1.280	6	1	K.LSKDFVEDISK.L (Ions score 48)
175	185	428	1.280	1.280	9	1	K.LSKDFVEDISK.L (Ions score 48)
175	193	742	2.224	2.224	7	2	K.LSKDFVEDISKLNIFELSK.G (Ions score 38)
175	193	742	2.224	2.224	8	2	K.LSKDFVEDISKLNIFELSK.G (Ions score 38)
178	185	477	951	951	2	0	K.DFVEDISK.L (Ions score 29)
178	185	477	951	951	7	0	K.DFVEDISK.L (Ions score 43)
178	185	477	951	951	8	0	K.DFVEDISK.L (Ions score 41)
178	185	477	951	951	9	0	K.DFVEDISK.L (Ions score 42)

178	193	633	1.896	1.896	4	1	K.DFVEDISKLNIFELSK.G (Ions score 65)
178	193	633	1.896	1.896	8	1	K.DFVEDISKLNIFELSK.G (Ions score 46)
178	193	633	1.896	1.896	8	1	K.DFVEDISKLNIFELSK.G (Ions score 31)
178	197	787	2.359	2.359	9	2	K.DFVEDISKLNIFELSKGYDK.K (Ions score 30)
186	193	482	963	963	5	0	K.LNIFELSK.G (Ions score 23)
186	193	482	963	963	6	0	K.LNIFELSK.G (Ions score 30)
186	193	482	963	963	8	0	K.LNIFELSK.G (Ions score 34)
186	197	476	1.426	1.426	6	1	K.LNIFELSKGYDK.K (Ions score 35)
186	198	519	1.554	1.554	8	2	K.LNIFELSKGYDKK.V (Ions score 19)
194	213	760	2.277	2.277	-6	2	K.GYDKKVLIVHGTNDEAVEYK.V (Ions score 36)
198	213	606	1.814	1.814	3	1	K.KVLIVHGTNDEAVEYK.V (Ions score 21)
198	213	606	1.814	1.814	3	1	K.KVLIVHGTNDEAVEYK.V (Ions score 24)
198	213	606	1.814	1.814	5	1	K.KVLIVHGTNDEAVEYK.V (Ions score 22)
198	213	606	1.814	1.814	5	1	K.KVLIVHGTNDEAVEYK.V (Ions score 20)
198	213	606	1.814	1.814	8	1	K.KVLIVHGTNDEAVEYK.V (Ions score 33)
198	213	606	1.814	1.814	8	1	K.KVLIVHGTNDEAVEYK.V (Ions score 40)
198	217	758	2.271	2.271	4	2	K.KVLIVHGTNDEAVEYKVSDR.I (Ions score 31)
198	217	758	2.271	2.271	5	2	K.KVLIVHGTNDEAVEYKVSDR.I (Ions score 59)
198	217	768	2.300	2.300	-8	2	K.KVLIVHGTNDEAVEYKVSDR.I Azidohomoalanin red. (A) (Ions score 8)
199	213	563	1.686	1.686	1	0	K.VLIVHGTNDEAVEYK.V (Ions score 59)
199	213	563	1.686	1.686	2	0	K.VLIVHGTNDEAVEYK.V (Ions score 11)
199	213	563	1.686	1.686	4	0	K.VLIVHGTNDEAVEYK.V (Ions score 56)
199	213	563	1.686	1.686	6	0	K.VLIVHGTNDEAVEYK.V (Ions score 19)
199	213	563	1.686	1.686	6	0	K.VLIVHGTNDEAVEYK.V (Ions score 46)
199	213	563	1.686	1.686	6	0	K.VLIVHGTNDEAVEYK.V (Ions score 5)
199	213	563	1.686	1.686	7	0	K.VLIVHGTNDEAVEYK.V (Ions score 10)
199	213	563	1.686	1.686	7	0	K.VLIVHGTNDEAVEYK.V (Ions score 37)
199	213	563	1.686	1.686	8	0	K.VLIVHGTNDEAVEYK.V (Ions score 26)
199	213	563	1.686	1.686	8	0	K.VLIVHGTNDEAVEYK.V (Ions score 60)
199	213	563	1.686	1.686	8	0	K.VLIVHGTNDEAVEYK.V (Ions score 52)

199	213	563	1.686	1.686	8	0	K.VLIVHGTNDEAVEYK.V (Ions score 55)
199	213	563	1.686	1.686	8	0	K.VLIVHGTNDEAVEYK.V (Ions score 32)
199	213	563	1.686	1.686	9	0	K.VLIVHGTNDEAVEYK.V (Ions score 45)
199	213	563	1.686	1.686	9	0	K.VLIVHGTNDEAVEYK.V (Ions score 37)
199	213	563	1.686	1.686	9	0	K.VLIVHGTNDEAVEYK.V (Ions score 30)
199	213	563	1.686	1.686	9	0	K.VLIVHGTNDEAVEYK.V (Ions score 26)
199	213	563	1.686	1.686	9	0	K.VLIVHGTNDEAVEYK.V (Ions score 62)
199	213	563	1.686	1.686	10	0	K.VLIVHGTNDEAVEYK.V (Ions score 49)
199	213	563	1.686	1.686	10	0	K.VLIVHGTNDEAVEYK.V (Ions score 48)
199	217	1.073	2.143	2.143	2	1	K.VLIVHGTNDEAVEYKVSDR.I (Ions score 79)
199	217	715	2.143	2.143	2	1	K.VLIVHGTNDEAVEYKVSDR.I (Ions score 59)
199	217	715	2.143	2.143	4	1	K.VLIVHGTNDEAVEYKVSDR.I (Ions score 3)
199	217	715	2.143	2.143	5	1	K.VLIVHGTNDEAVEYKVSDR.I (Ions score 24)
199	217	715	2.143	2.143	5	1	K.VLIVHGTNDEAVEYKVSDR.I (Ions score 24)
199	217	715	2.143	2.143	6	1	K.VLIVHGTNDEAVEYKVSDR.I (Ions score 38)
199	217	715	2.143	2.143	7	1	K.VLIVHGTNDEAVEYKVSDR.I (Ions score 25)
199	217	715	2.143	2.143	7	1	K.VLIVHGTNDEAVEYKVSDR.I (Ions score 45)
199	217	715	2.143	2.143	7	1	K.VLIVHGTNDEAVEYKVSDR.I (Ions score 32)
199	217	715	2.143	2.143	7	1	K.VLIVHGTNDEAVEYKVSDR.I (Ions score 47)
199	217	715	2.143	2.143	7	1	K.VLIVHGTNDEAVEYKVSDR.I (Ions score 42)
199	217	715	2.143	2.143	7	1	K.VLIVHGTNDEAVEYKVSDR.I (Ions score 51)
199	217	715	2.143	2.143	8	1	K.VLIVHGTNDEAVEYKVSDR.I (Ions score 42)
199	217	715	2.143	2.143	8	1	K.VLIVHGTNDEAVEYKVSDR.I (Ions score 22)
199	217	715	2.143	2.143	8	1	K.VLIVHGTNDEAVEYKVSDR.I (Ions score 24)
199	217	715	2.143	2.143	9	1	K.VLIVHGTNDEAVEYKVSDR.I (Ions score 46)
199	217	725	2.172	2.172	-9	1	K.VLIVHGTNDEAVEYKVSDR.I Azidohomoalanin red. (A) (Ions score 12)
199	220	833	2.497	2.497	9	2	K.VLIVHGTNDEAVEYKVSDRILK.E (Ions score 21)
214	220	416	830	830	8	1	K.VSDRILK.E (Ions score 20)
214	229	613	1.835	1.835	2	2	K.VSDRILKEVYGDNATR.V (Ions score 41)
218	229	460	1.378	1.378	0	1	R.ILKEVYGDNATR.V (Ions score 27)

218	229	460	1.378	1.378	4	1	R.ILKEVYGDNATR.V (Ions score 47)
218	229	460	1.378	1.378	5	1	R.ILKEVYGDNATR.V (Ions score 18)
218	229	460	1.378	1.378	6	1	R.ILKEVYGDNATR.V (Ions score 6)
218	229	460	1.378	1.378	7	1	R.ILKEVYGDNATR.V (Ions score 9)
218	229	460	1.378	1.378	8	1	R.ILKEVYGDNATR.V (Ions score 42)
218	229	460	1.378	1.378	8	1	R.ILKEVYGDNATR.V (Ions score 13)
218	229	460	1.378	1.378	8	1	R.ILKEVYGDNATR.V (Ions score 8)
218	240	879	2.633	2.633	4	2	R.ILKEVYGDNATRVTIENADHTFK.S (Ions score 27)
221	229	513	1.023	1.023	8	0	K.EVYGDNATR.V (Ions score 45)
221	240	761	2.279	2.279	-2	1	K.EVYGDNATRVTIENADHTFK.S (Ions score 37)
221	240	761	2.279	2.279	5	1	K.EVYGDNATRVTIENADHTFK.S (Ions score 41)
230	240	638	1.274	1.274	1	0	R.VTIENADHTFK.S (Ions score 39)
230	240	426	1.274	1.274	6	0	R.VTIENADHTFK.S (Ions score 24)
230	240	426	1.274	1.274	6	0	R.VTIENADHTFK.S (Ions score 31)
230	240	426	1.274	1.274	6	0	R.VTIENADHTFK.S (Ions score 43)
230	240	426	1.274	1.274	9	0	R.VTIENADHTFK.S (Ions score 15)
230	246	683	2.046	2.046	2	1	R.VTIENADHTFKSLEWEK.K (Ions score 17)
230	246	683	2.046	2.046	7	1	R.VTIENADHTFKSLEWEK.K (Ions score 35)
230	246	683	2.046	2.046	8	1	R.VTIENADHTFKSLEWEK.K (Ions score 50)
230	247	726	2.174	2.174	8	2	R.VTIENADHTFKSLEWEKK.A (Ions score 28)
241	246	396	790	790	6	0	K.SLEWEK.K (Ions score 32)
241	246	396	790	790	9	0	K.SLEWEK.K (Ions score 23)
241	247	460	918	918	7	1	K.SLEWEKK.A (Ions score 27)
241	247	460	918	918	7	1	K.SLEWEKK.A (Ions score 26)
241	247	460	918	918	8	1	K.SLEWEKK.A (Ions score 24)
247	257	443	1.326	1.326	9	1	K.KAIEESVEFFK.K (Ions score 28)
247	258	486	1.454	1.454	6	2	K.KAIEESVEFFKK.E (Ions score 63)
248	257	600	1.198	1.198	7	0	K.AIEESVEFFK.K (Ions score 64)
248	258	664	1.326	1.326	6	1	K.AIEESVEFFKK.E (Ions score 9)
248	258	664	1.326	1.326	6	1	K.AIEESVEFFKK.E (Ions score 56)

248	258	443	1.326	1.326	7	1	K.AIEESVEFFKK.E (Ions score 32)
248	258	443	1.326	1.326	7	1	K.AIEESVEFFKK.E (Ions score 15)
248	258	443	1.326	1.326	8	1	K.AIEESVEFFKK.E (Ions score 25)
248	262	604	1.809	1.809	-8	2	K.AIEESVEFFKKELLK.G (Ions score 16)
263	279	666	1.994	1.994	7	0	K.GGSENLYFQSAHHHHHH.- (Ions score 27)

Lipase activity test

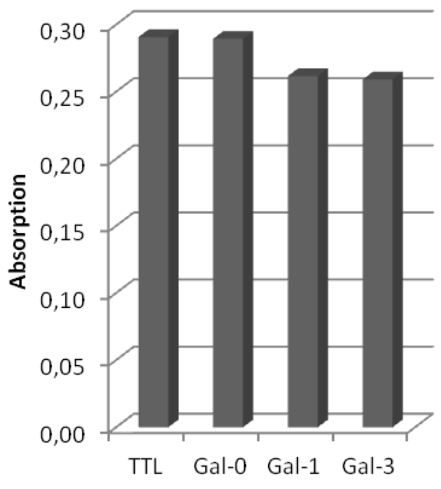

Figure S10: Lipase activity probed by the ester cleavage of 4-nitrophenyl palmitate.

Surface-plasmon-resonance (SPR)

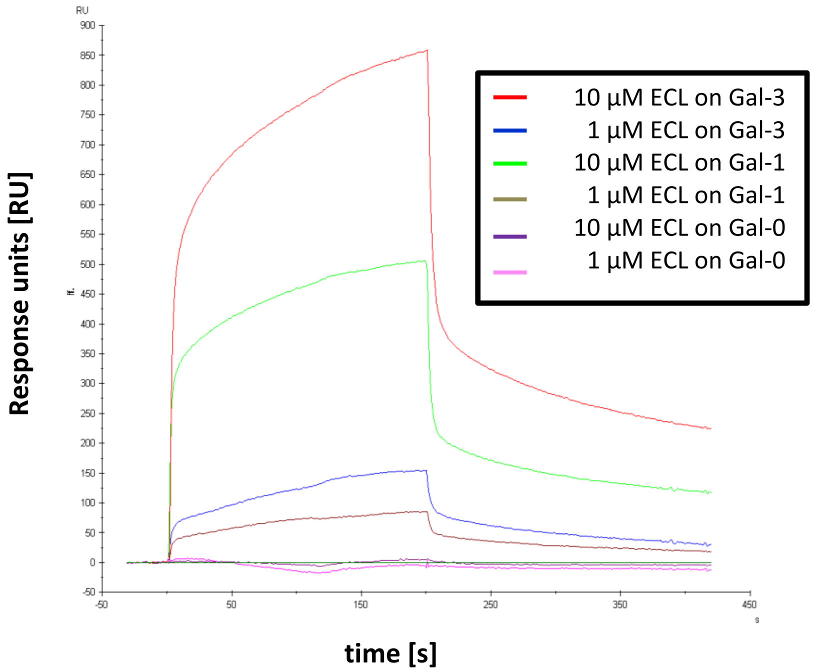

Figure S11: Overlayed sensorgram for initial ECL binding study (chips fully loaded).

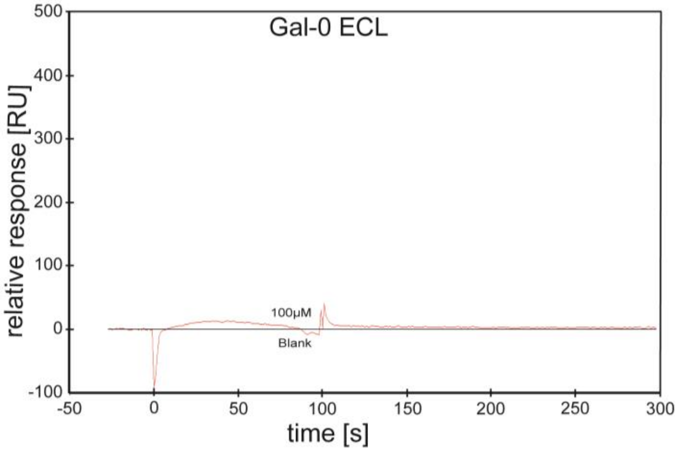

Figure S12: Overlayed sensorgrams from KD-measurements for **Gal-0**.

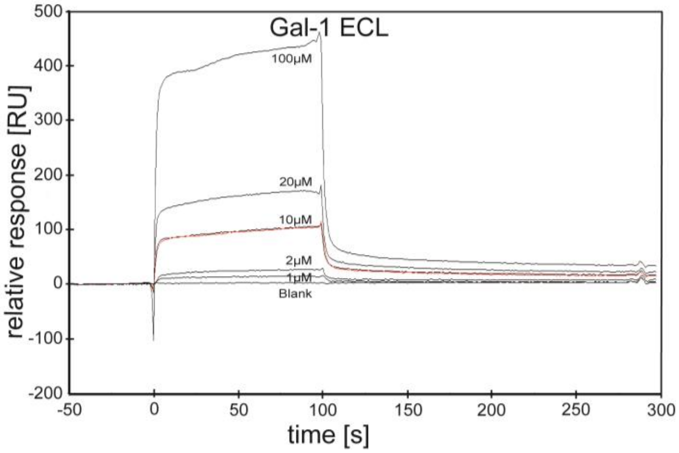

Figure S13: Overlayed sensorgrams from K_D-measurements for **Gal-1**. The 10 µM concentration was measured twice.

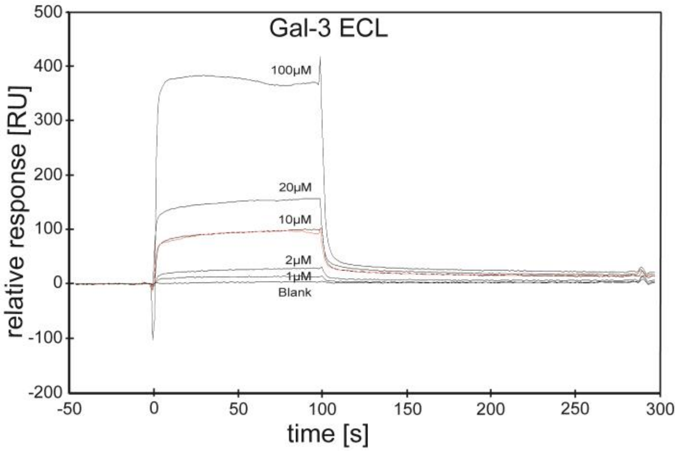

Figure S14: Overlayed sensorgrams from K_D-measurements for **Gal-3**. The 10 µM concentration was measured twice.

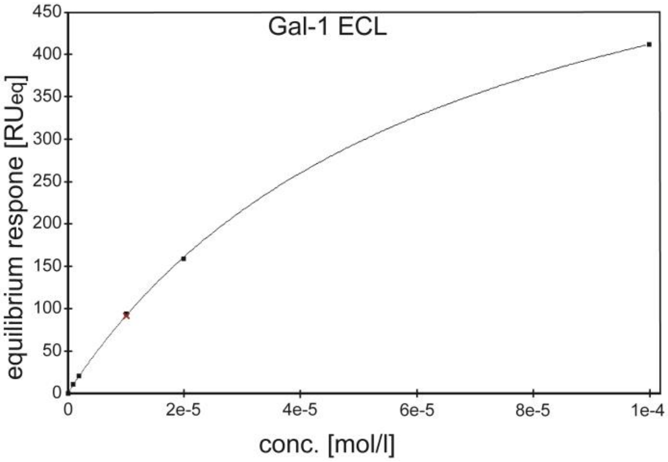

Figure S15: Langmuir binding isotherm of **Gal-1**.

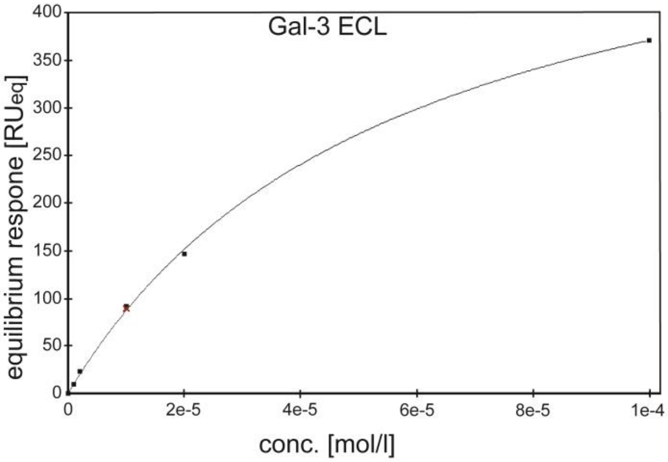

Figure S16: Langmuir binding isotherm of **Gal-3**.

References

[1] V. Hong *et al.* , *Angew. Chem. Int. Ed.*, **2009**, *48*, 9879.

[2] L. Merkel, Y. Cheburkin, B. Wiltschi, N. Budisa, *ChemBioChem* **2007**, *8*, 2227.

[3] M.G. Hoesl, N. Budisa, *ChemBioChem* **2011**, *12*, 552.

[4] L. Merkel, Y. Cheburkin, B. Wiltschi, N. Budisa, *ChemBioChem* **2007**, *8*, 2227.

[5] M.G. Hoesl, C.G. Acevedo-Rocha, S. Nehring, M. Royter, C. Wolschner, B. Wiltschi, N. Budisa, G. Antranikian, *ChemCatChem* **2011**, *3*, 213.

[6] N. Budisa, B. Steipe, P. Demange, C. Eckerskorn, J. Kellermann, R. Huber, *Eur. J. Biochem.* **1995**, *230*, 788.

[7] T.-P. Wang *et al.*, *Bioconjugate Chem.* **2010**, *21*, 1642.

[8] C. Gnaccarini *et al.*, *Bioorg. & Med. Chem.* **2009**, *17*, 6354.

[9] X. Li *et al.*, *J. Am. Chem. Soc.* **2011**, *133*, 11147.

[10] H. Staudt, M. G. Hoesl, A. Dreuw, S. Serdjukow, D. Oesterhelt, N. Budisa, J. Wachtveit, M. Grininger, *Angew. Chem. Int. Ed.* **2013**, *52*, 8463-8466.
